# Supplementary material for: Biological and Cheminformatics Studies of Newly Designed Triazole Based Derivatives as Potent Inhibitors against Mushroom Tyrosinase
Source: Molecules. 2022 Mar 7;27(5):1731. doi: 10.3390/molecules27051731 (PMC8911699; doi:10.3390/molecules27051731)
Supplement: Supplementary file 1 [file molecules-27-01731-s001.zip › molecules-1618436-supplementary.pdf]

Supporting Information

Biological and cheminformatics studies of newly designed tri-azole based derivatives as potent inhibitors against mushroom tyrosinase

Mubashir Hassan[1,2], Balasaheb D. Vanjare[3], Kyou-Yeong Sim[3], Hussain Raza[4], Ki Hwan Lee [3]*, Saba Shahzadi[5], Andrzej Kloczkowski[2,6]*

[1] Institute of Molecular Biology and Biotechnology, The University of Lahore-54590, Pakistan

[2] Battelle Center for Mathematical Medicine, The Research Institute at Nationwide Children's Hospital, Columbus, Ohio 43205, USA.

[3] Department of Chemistry, Kongju National University, Gongju, Chungnam-do 32588, Republic of Korea.

[4] Department of Biological Science, Kongju National University, Gongju, Chungnam-do 32588, Republic of Korea.

[5] Institute of Molecular Sciences and Bioinformatics, Nesbit Road Lahore, Pakistan

[6] Department of Pediatrics, The Ohio State University, Columbus, Ohio 43205, USA

Corresponding Authors:

Prof. Andrzej Kloczkowski,

Battelle Center for Mathematical Medicine, The Research Institute at Nationwide Children's Hospital, Columbus, Ohio 43205, USA. Email: Andrzej.Kloczkowski@nationwidechildrens.org

Dr. Ki Hwan Lee,

Dept. of Chemistry, Kongju National University, Gongju, Chungnam do 32588, Republic of Korea. Email: khlee@kongju.ac.kr

Supporting Figure captions:

Figure S1, ES‡: ^{1}H NMR Spectrum of **9a**

Figure S2, ES‡: ^{13}C NMR Spectrum of **9a**

Figure S3, ES‡: IR Spectrum of **9a**

Figure S4, ES‡: LCMS Spectrum of **9a**

Figure S5, ES‡: ^{1}H NMR Spectrum of **9b**

Figure S6, ES‡: ^{13}C NMR Spectrum of **9b**

Figure S7, ES‡: IR Spectrum of **9b**

Figure S8, ES‡: LCMS Spectrum of **9b**

Figure S9, ES‡: ^{1}H NMR Spectrum of **9c**

Figure S10, ES‡: ^{13}C NMR Spectrum of **9c**

Figure S11, ES‡: IR Spectrum of **9c**

Figure S12, ES‡: LCMS Spectrum of **9c**

Figure S13, ES‡: ^{1}H NMR Spectrum of **9d**

Figure S14, ES‡: ^{13}C NMR Spectrum of **9d**

Figure S15, ES‡: IR Spectrum of **9d**

Figure S16, ES‡: LCMS Spectrum of **9d**

Figure S17, ES‡: ^{1}H NMR Spectrum of **9e**

Figure S18, ES‡: ^{13}C NMR Spectrum of **9e**

Figure S19, ES‡: IR Spectrum of **9e**

Figure S20, ES‡: LCMS Spectrum of **9e**

Figure S21, ES‡: ^{1}H NMR Spectrum of **9f**

Figure S22, ES‡: ^{13}C NMR Spectrum of **9f**

Figure S23, ES‡: IR Spectrum of **9f**

Figure S24, ES‡: LCMS Spectrum of **9f**

Figure S25, ES‡: ^{1}H NMR Spectrum of **9g**

Figure S26, ES‡: ^{13}C NMR Spectrum of **9g**

Figure S27, ES‡: IR Spectrum of **9g**

Figure S28, ES‡: LCMS Spectrum of **9g**

Figure S29, ES‡: ^{1}H NMR Spectrum of **9h**

Figure S30, ES‡: ^{13}C NMR Spectrum of **9h**

Figure S31, ES‡: IR Spectrum of **9h**

Figure S32, ES‡: LCMS Spectrum of **9h**

Figure S33, ES‡: ^{1}H NMR Spectrum of **9i**

Figure S34, ES‡: ^{13}C NMR Spectrum of **9i**

Figure S35, ES‡: IR Spectrum of **9i**

Figure S36, ES‡: LCMS Spectrum of **9i**

Figure S37, ES‡: The Mushroom tyrosinase Ramachandran graph is presented the residual position in the favor region (pink boundary) by evaluating the Psi (φ) and Phi (ψ) angles. Most of residues (green circle) lie in favor region while only ten poor rotamers are present in the outer region.

Figure S38, ES‡: Docking image of **9a** against mushroom tyrosinase

Figure S39, ES‡: Docking image of **9b** against mushroom tyrosinase

Figure S40, ES‡: Docking image of **9c** against mushroom tyrosinase

Figure S41, ES‡: Docking image of **9d** against mushroom tyrosinase

Figure S42, ES‡: Docking image of **9e** against mushroom tyrosinase

Figure S43, ES‡: Docking image of **9f** against mushroom tyrosinase

Figure S44, ES‡: Docking image of **9g** against mushroom tyrosinase

Figure S45, ES‡: Docking image of **9h** against mushroom tyrosinase

Figure S46, ES‡: Docking image of **9i** against mushroom tyrosinase

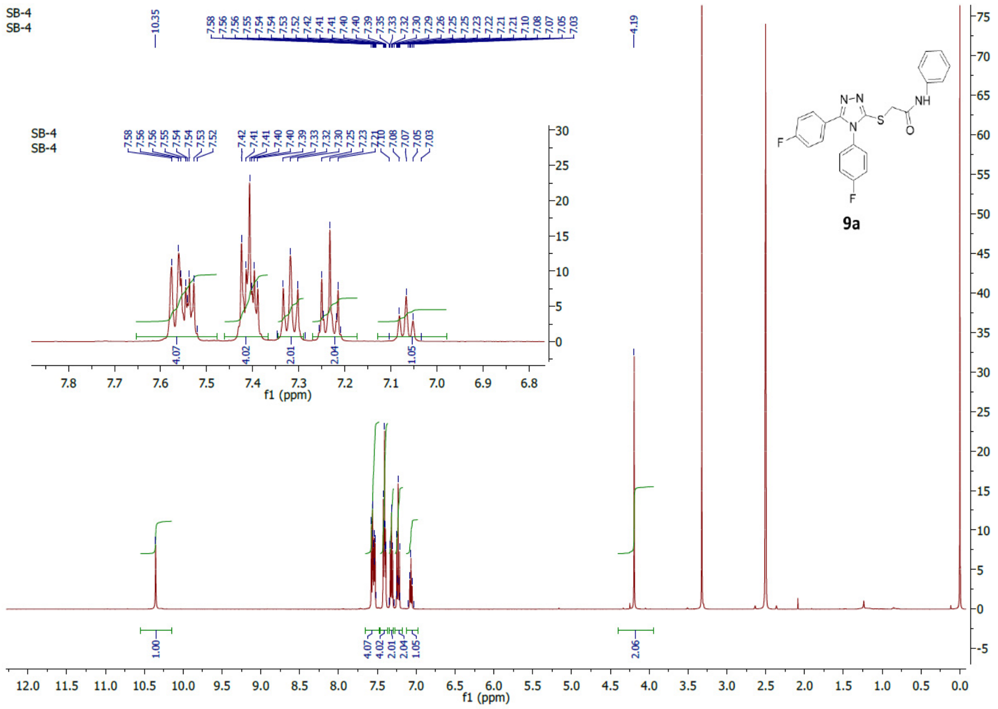

Figure S1, ES‡

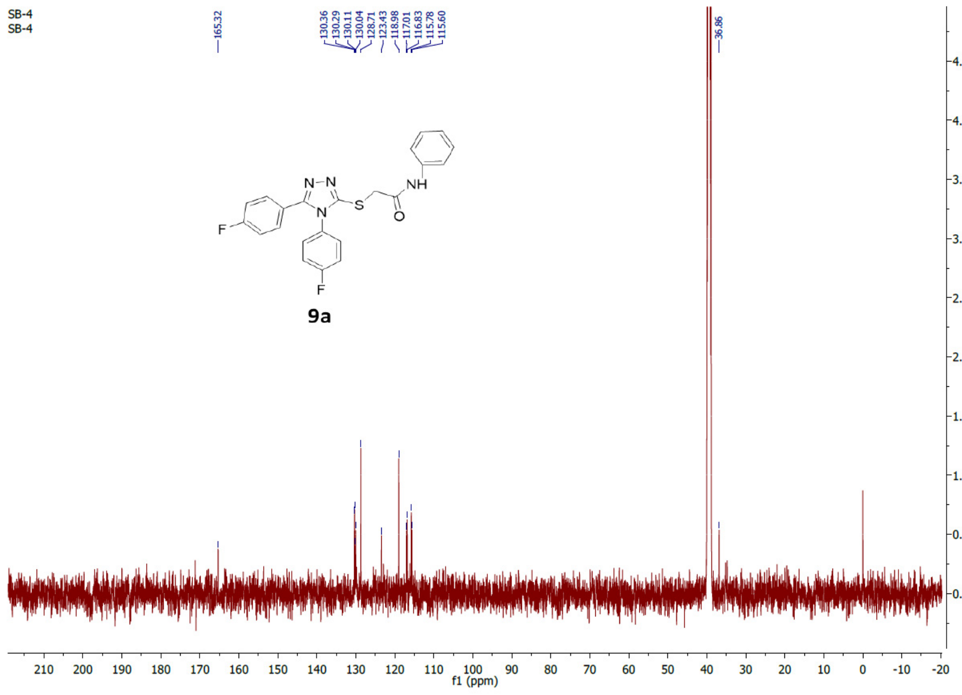

SB-4
SB-4
165.32
130.36
130.29
130.11
130.04
128.71
123.43
118.98
117.01
116.83
115.78
115.60
36.86
9a
f1 (ppm)

Figure S2, ES‡

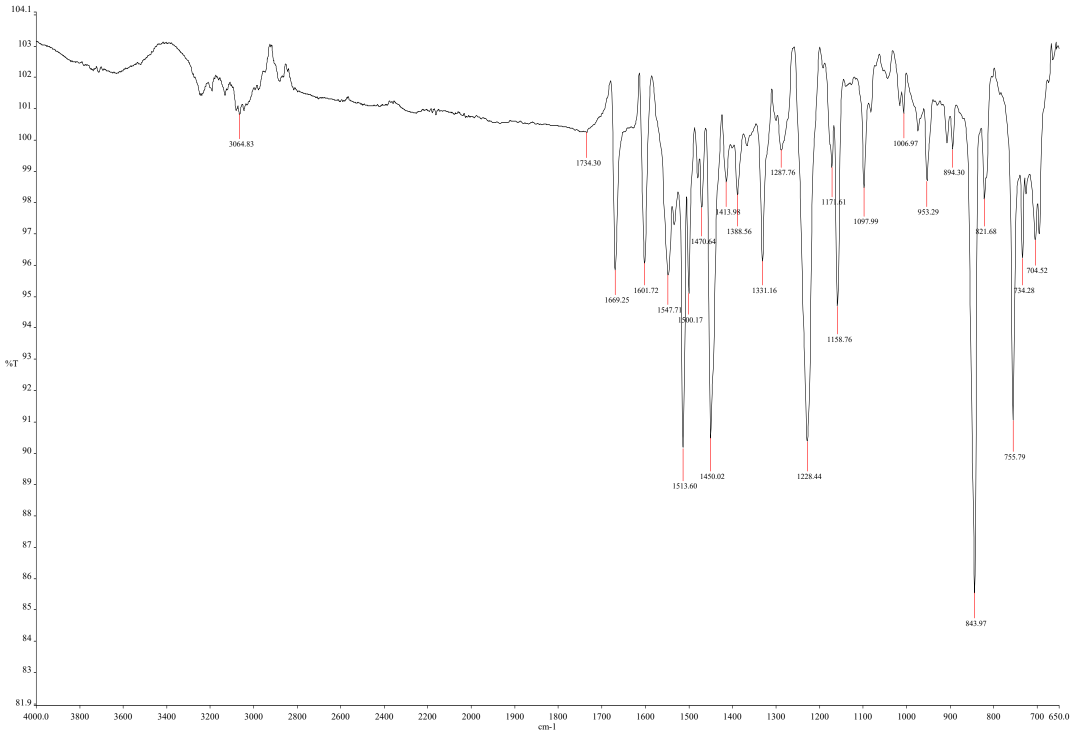

%T
cm-1
3064.83
1734.30
1669.25
1601.72
1547.71
1513.60
1500.17
1470.64
1450.02
1413.98
1388.56
1331.16
1287.76
1228.44
1171.61
1158.76
1097.99
1006.97
953.29
894.30
843.97
821.68
755.79
734.28
704.52

Figure S3, ES‡

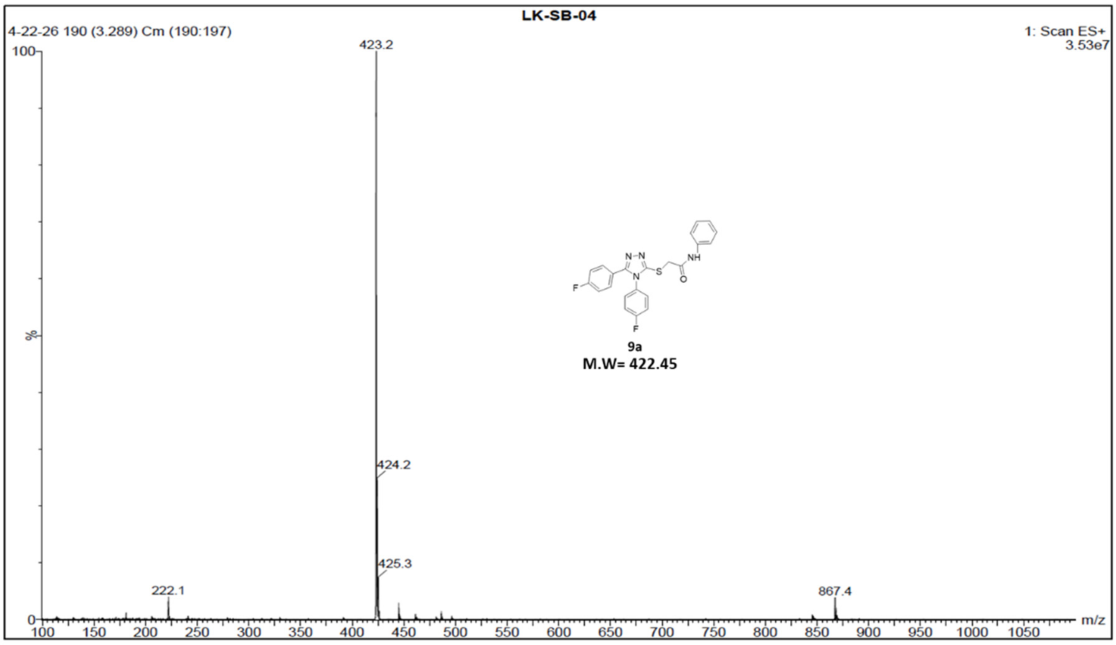

Figure S4, ES‡

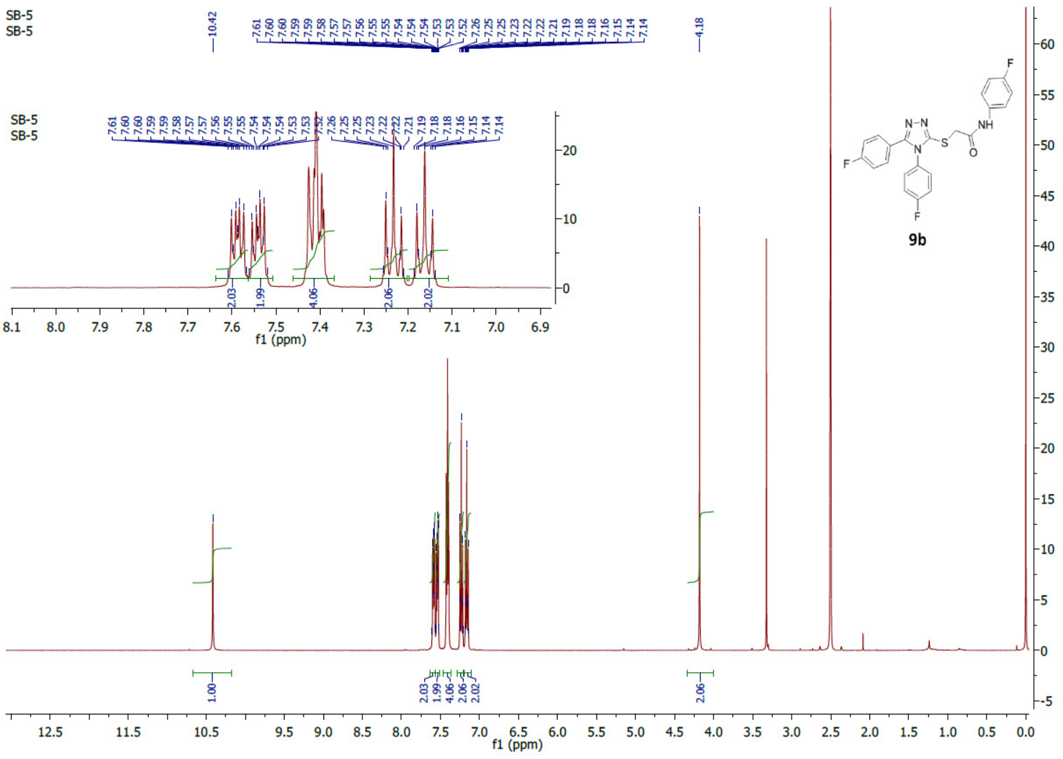

Figure S5, ES‡

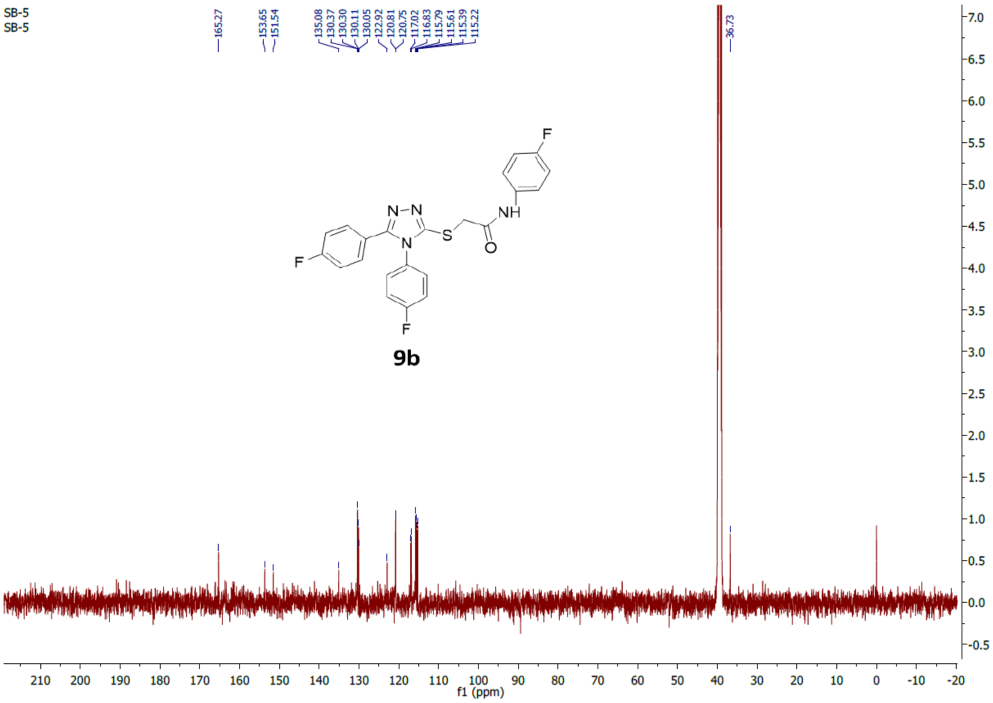

Figure S6, ES‡

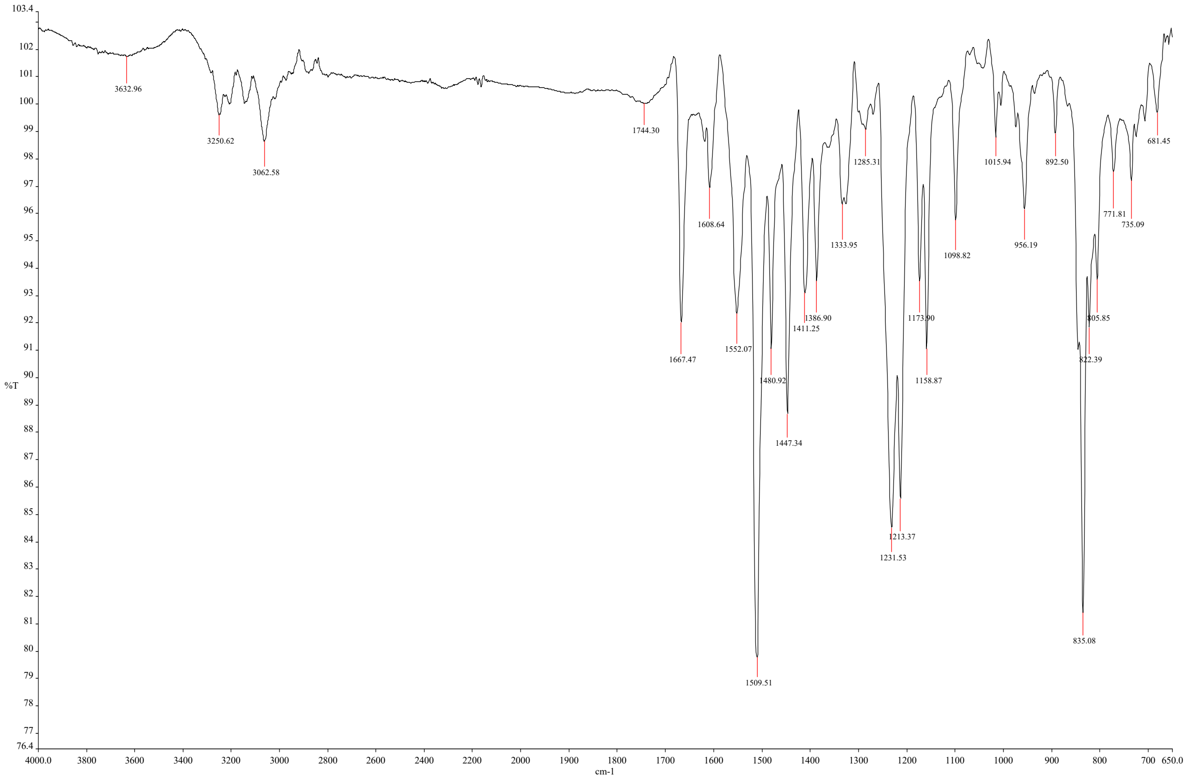

%T
cm-1
3632.96
3250.62
3062.58
1744.30
1667.47
1608.64
1552.07
1509.51
1480.92
1447.34
1411.25
1386.90
1333.95
1285.31
1231.53
1213.37
1173.90
1158.87
1098.82
1015.94
956.19
892.50
835.08
822.39
805.85
771.81
735.09
681.45

Figure S7, ES‡

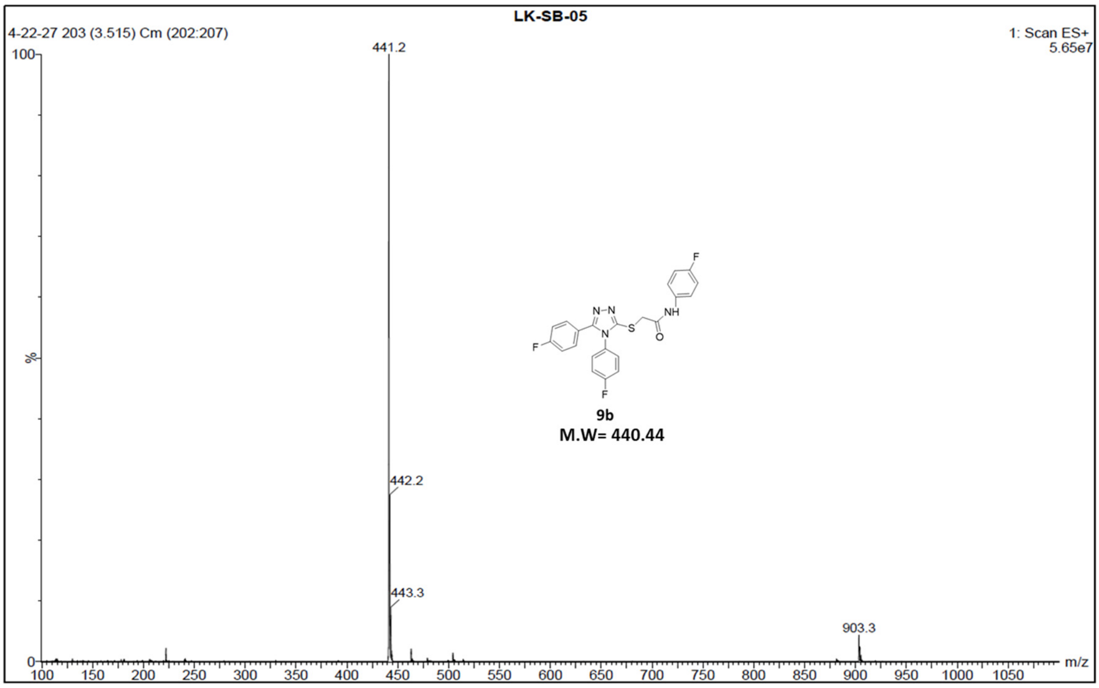

LK-SB-05
4-22-27 203 (3.515) Cm (202:207)
1: Scan ES+
5.65e7
441.2
442.2
443.3
903.3
%
m/z
9b
M.W= 440.44

Figure S8, ES‡

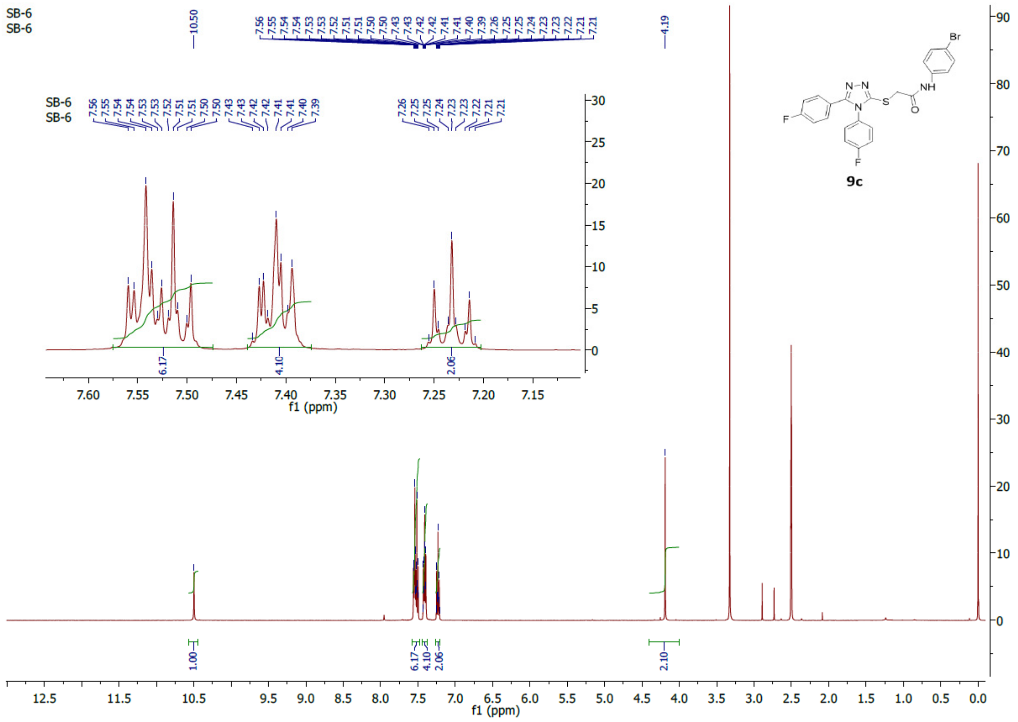

Figure S9, ES‡

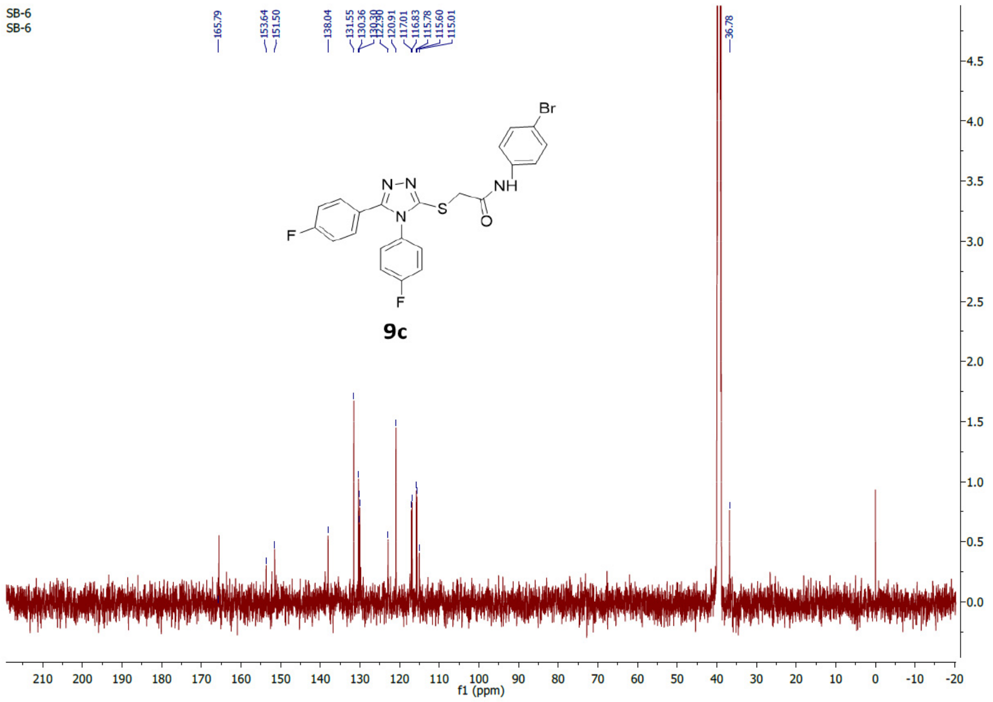

Figure S10, ES‡

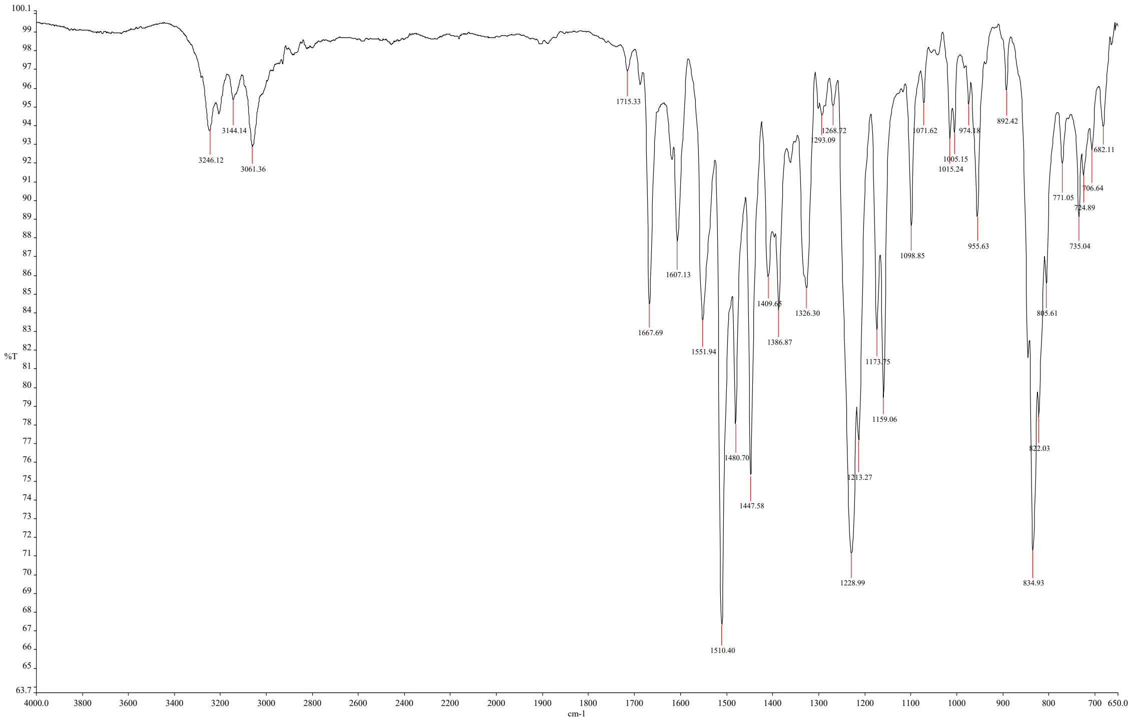

Figure S11, ES‡

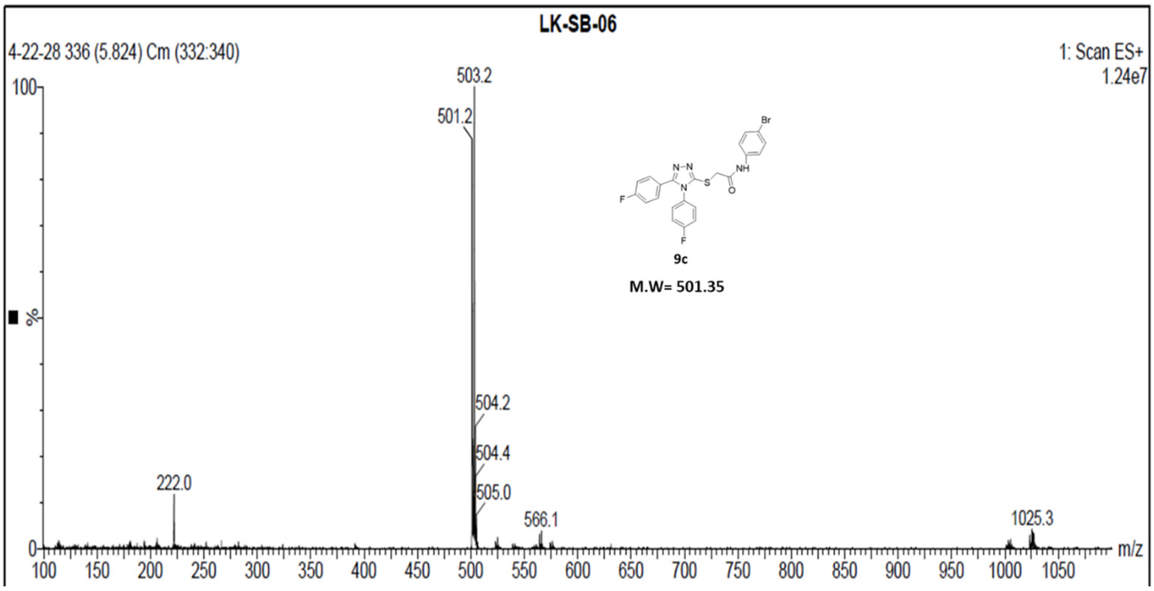

Figure S12, ES‡

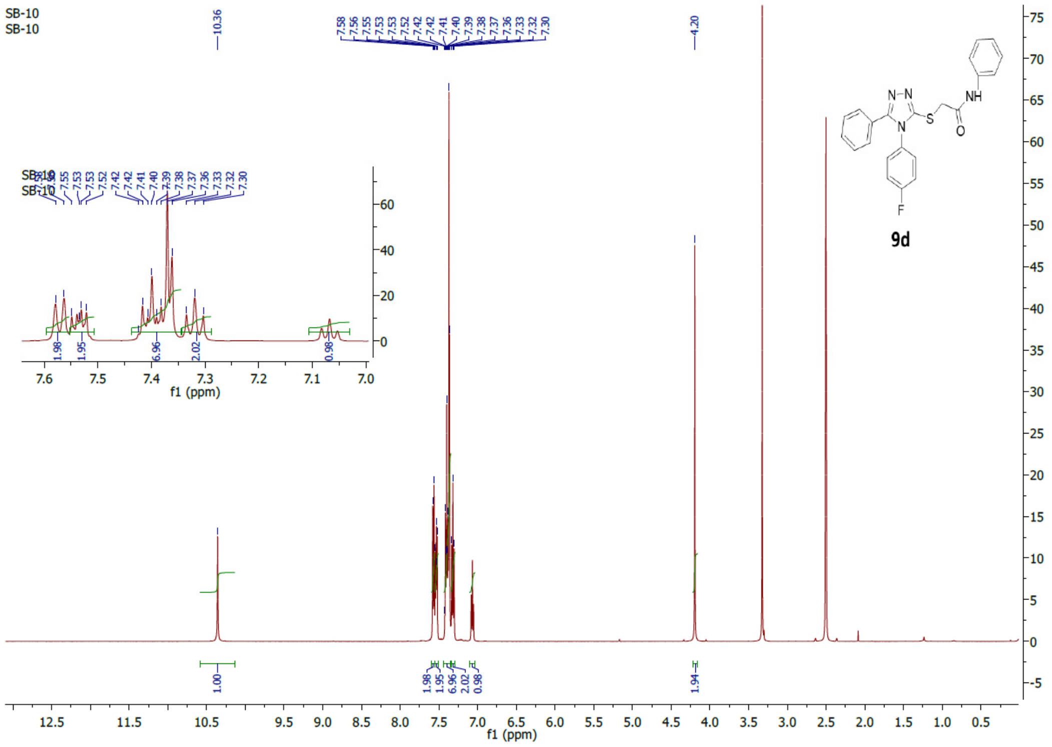

Figure S13, ES‡

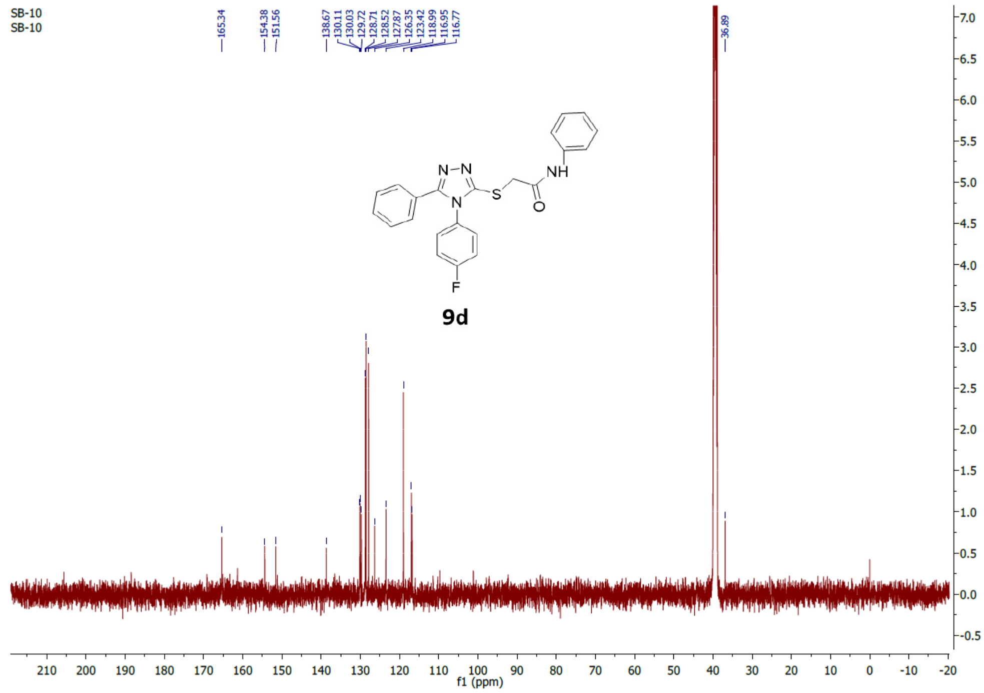

Figure S14, ES‡

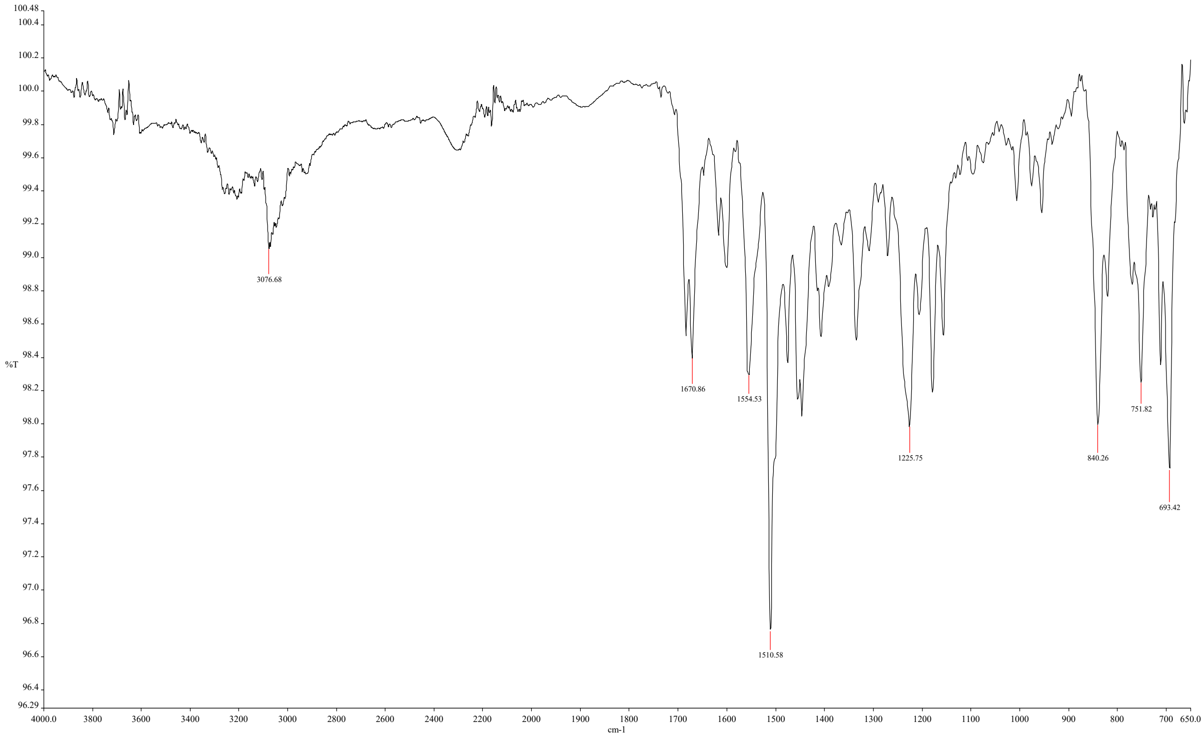

Figure S15, ES‡

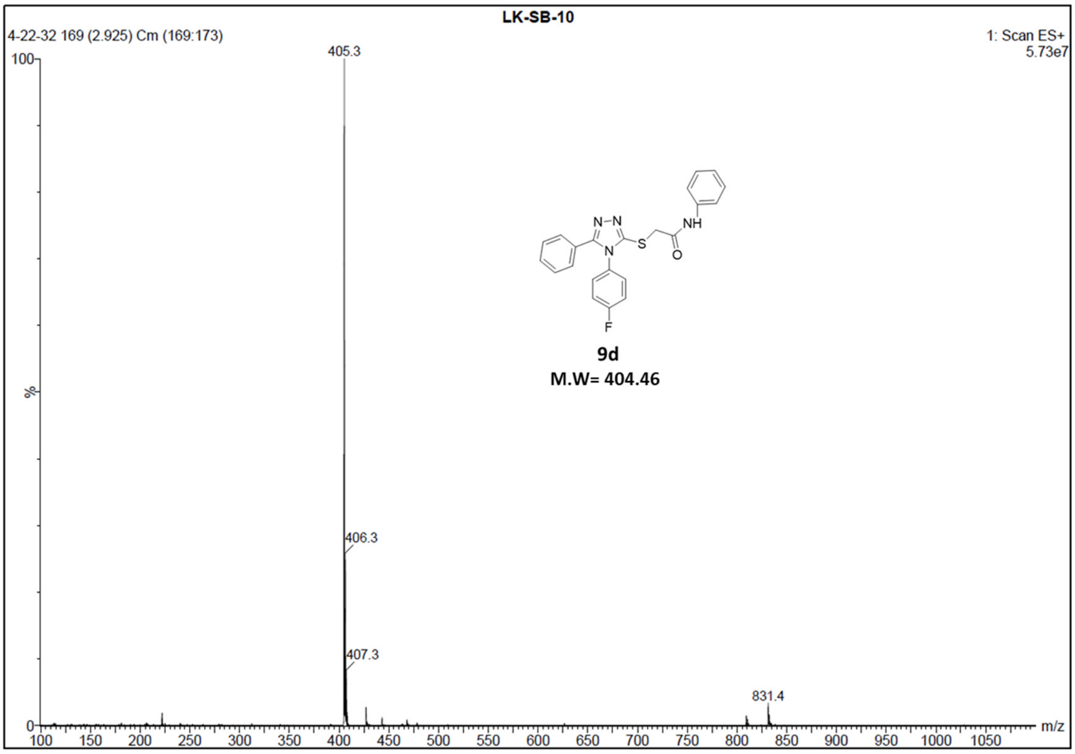

Figure S16, ES‡

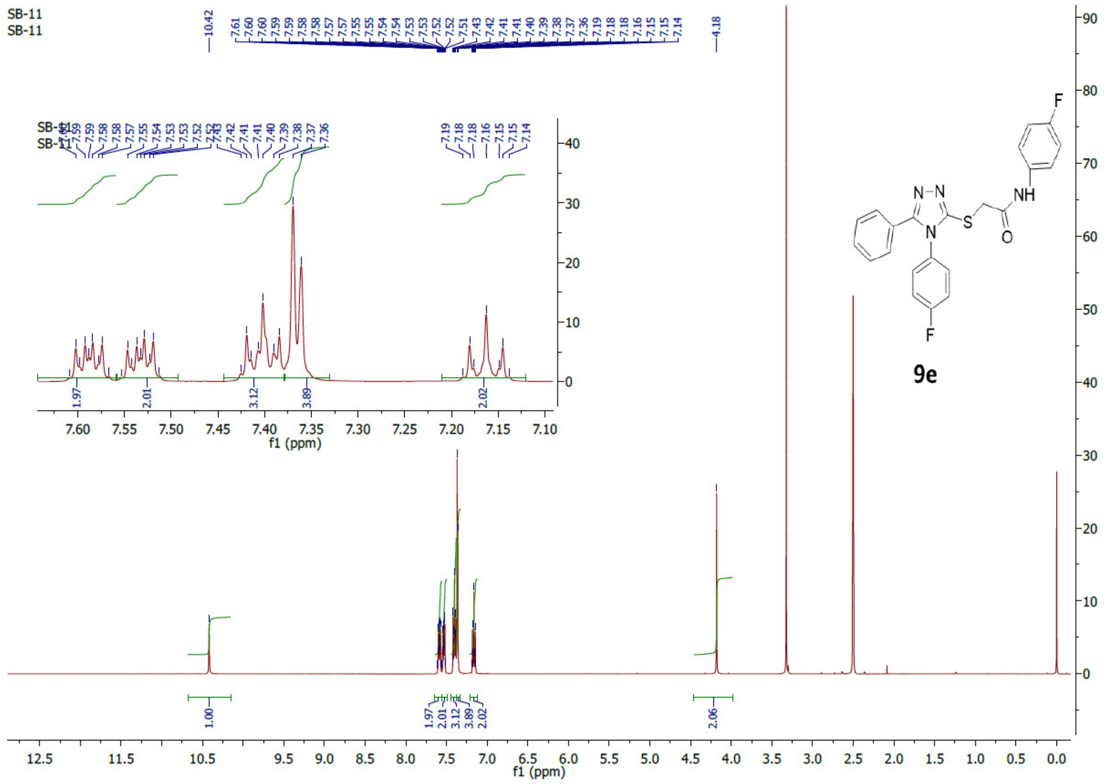

Figure S17, ES‡

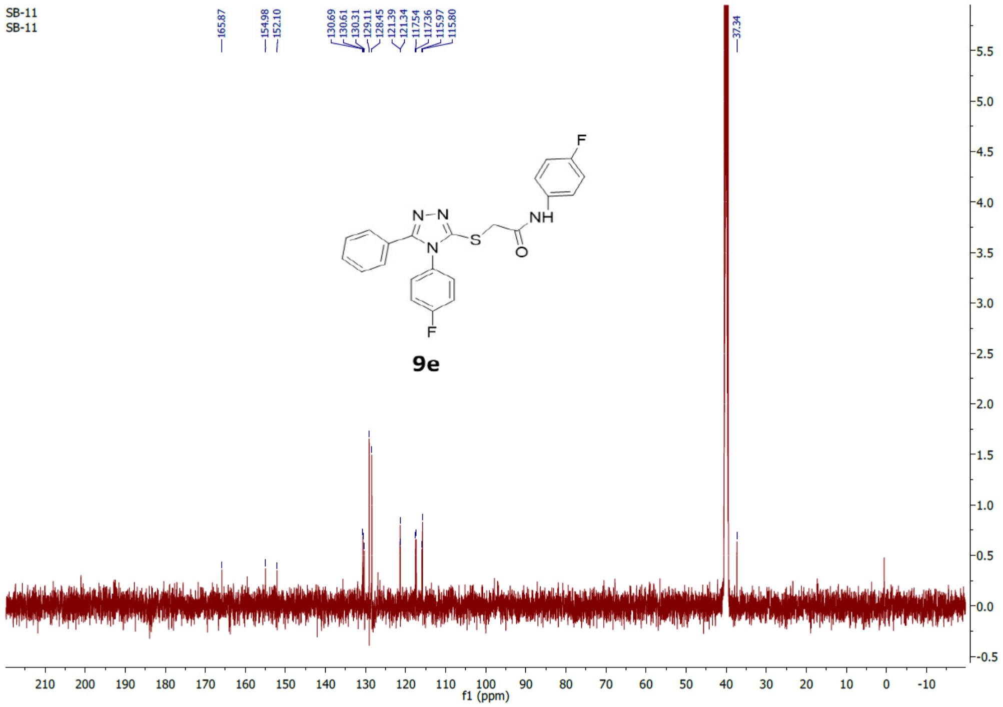

Figure S18, ES‡

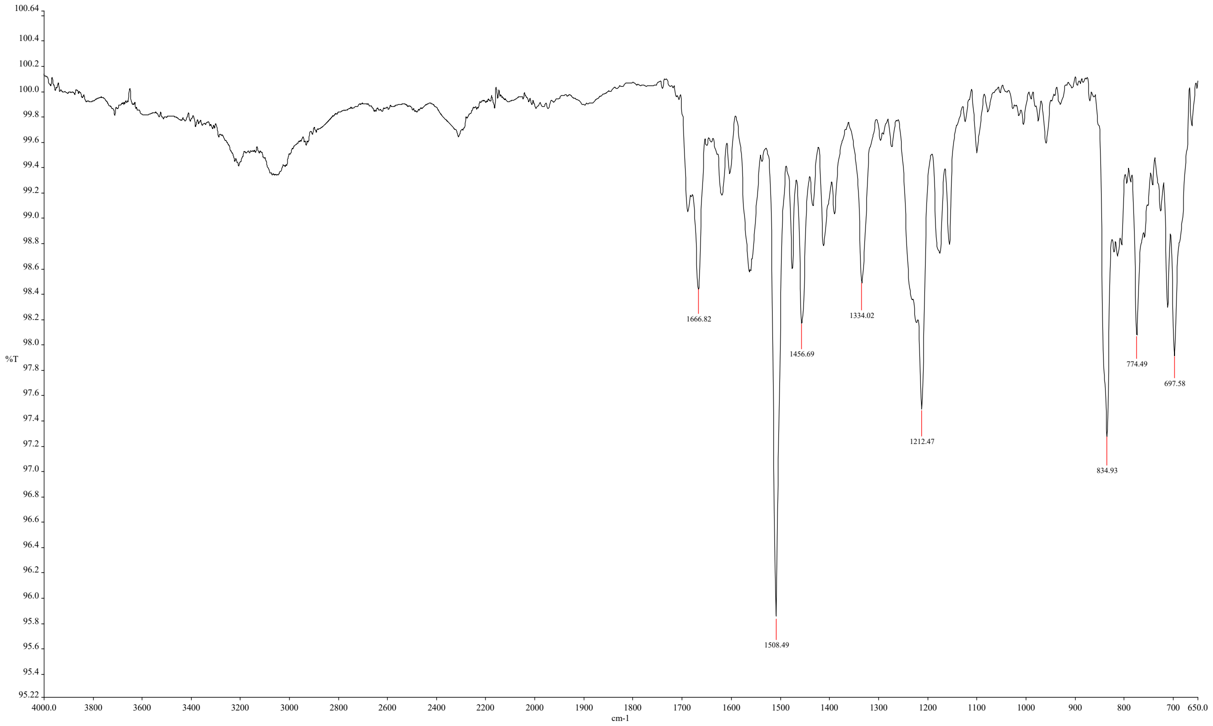

Figure S19, ES‡

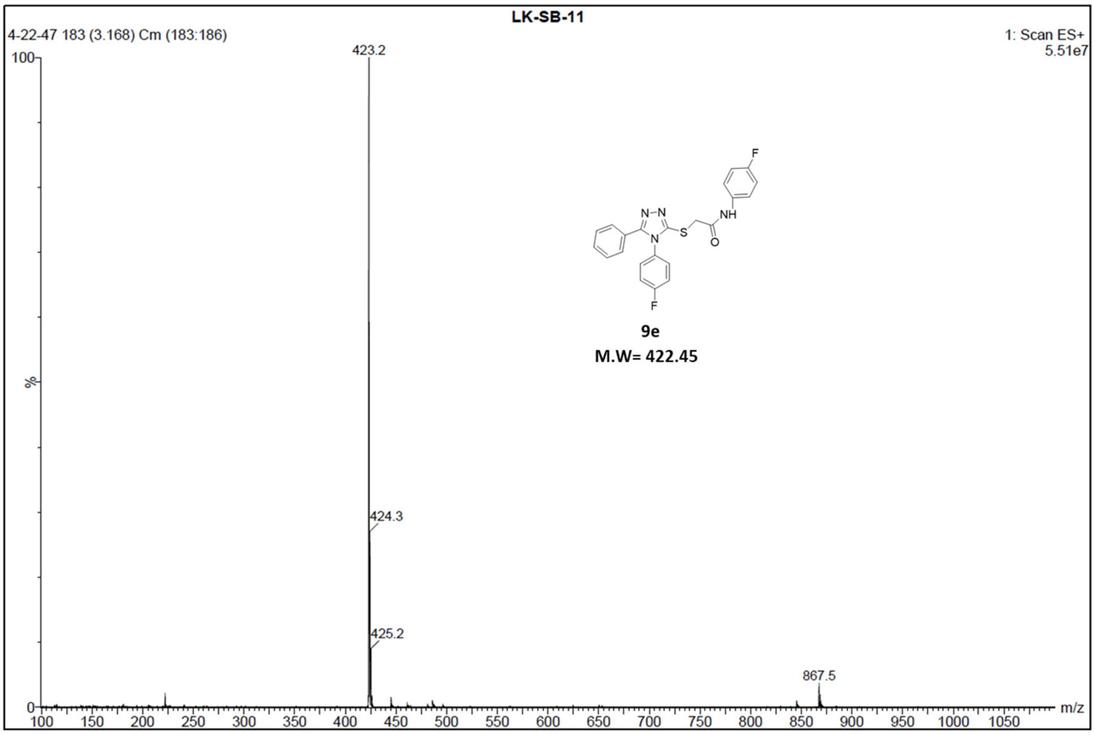

Figure S20, ES‡

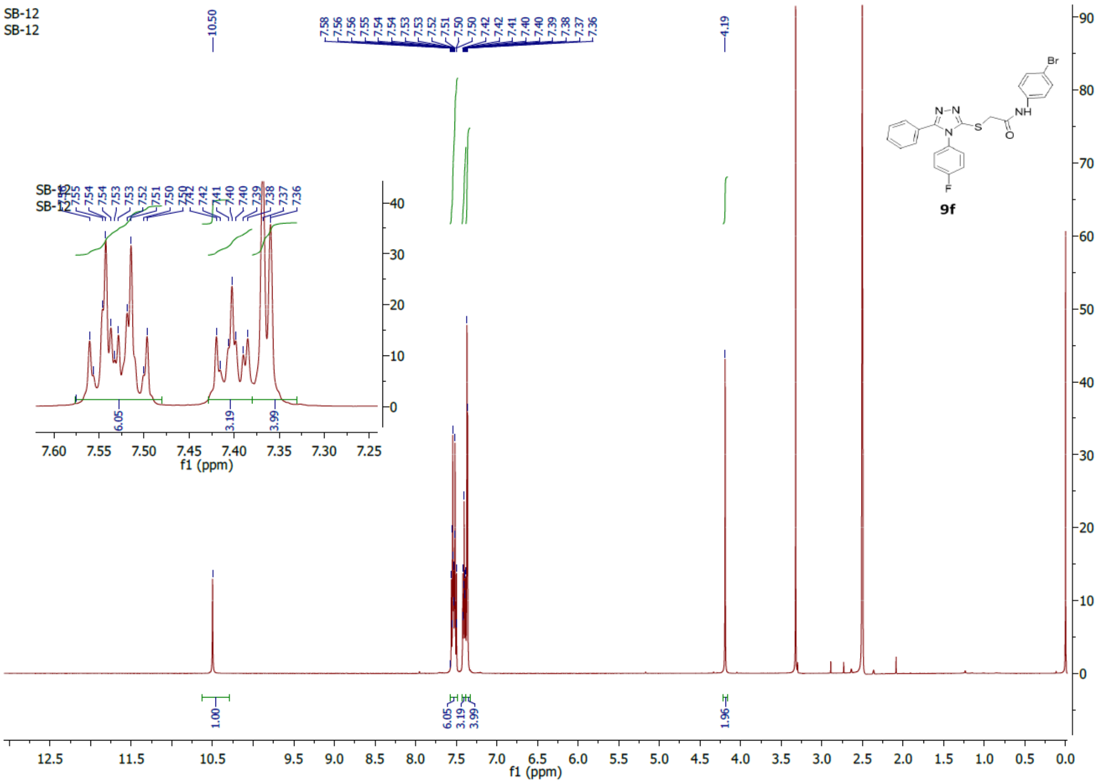

Figure S21, ES‡

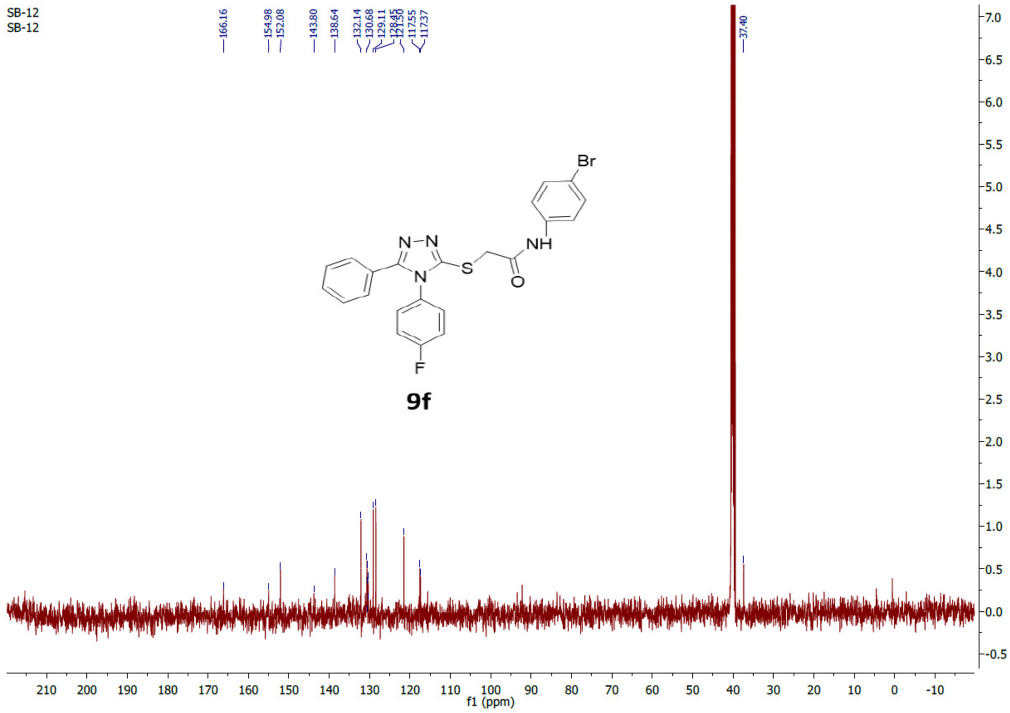

Figure S22, ES‡

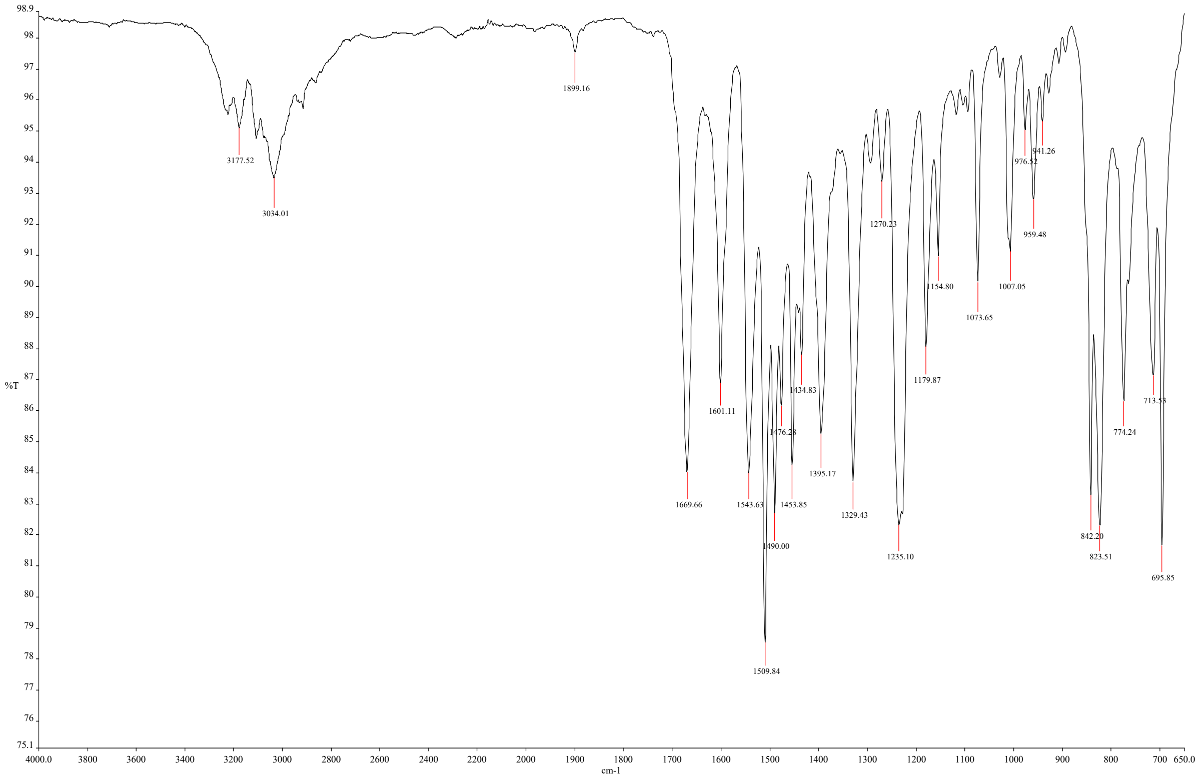

Figure S23, ES‡

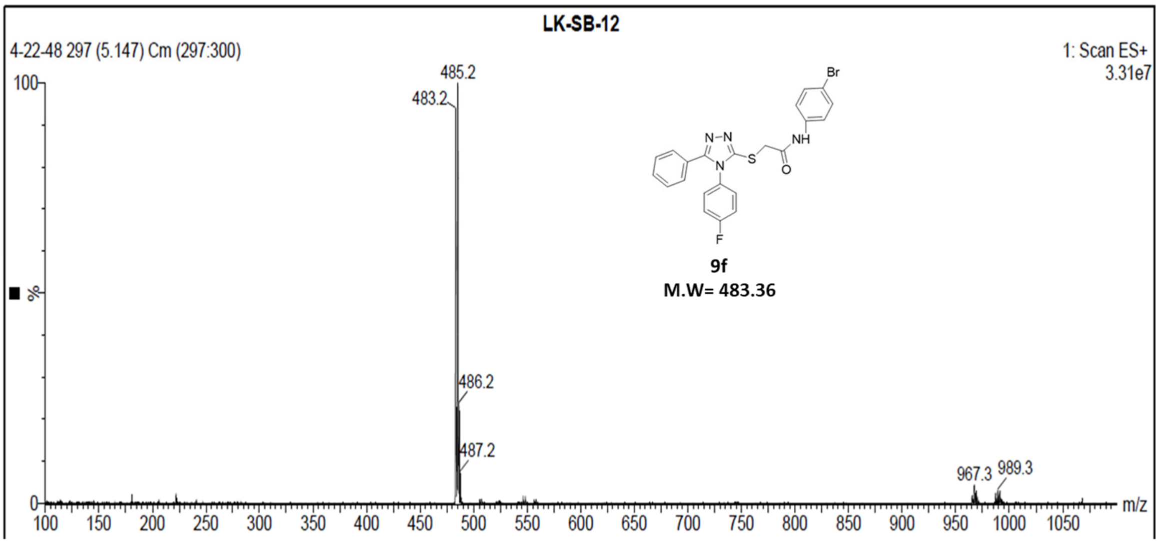

Figure S24, ES‡

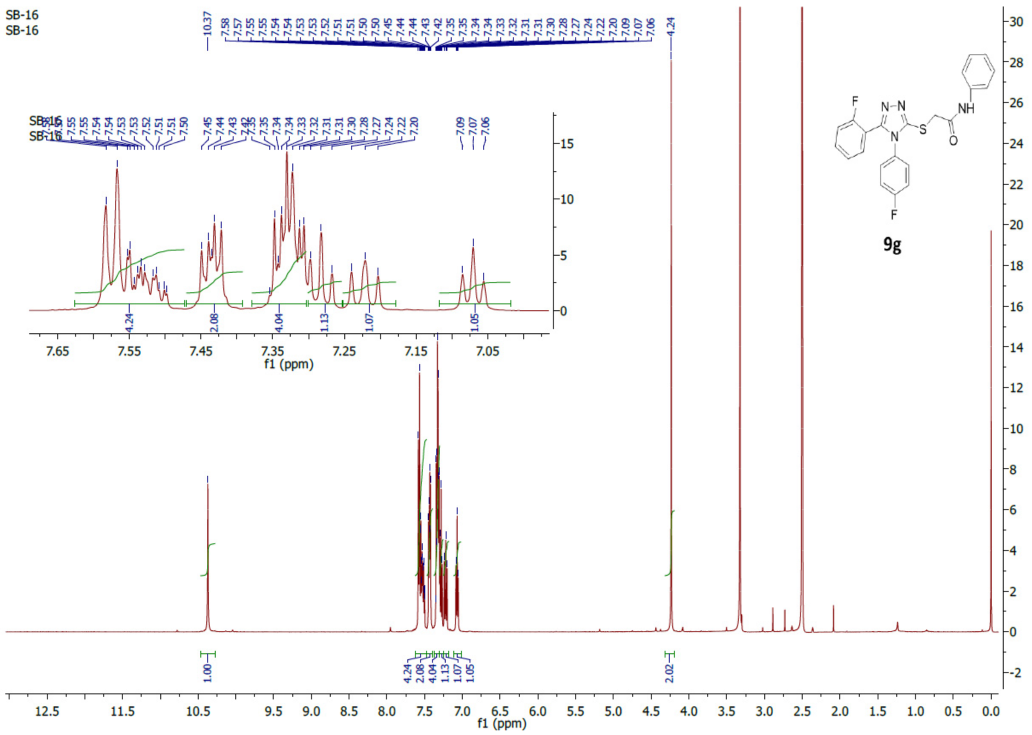

Figure S25, ES‡

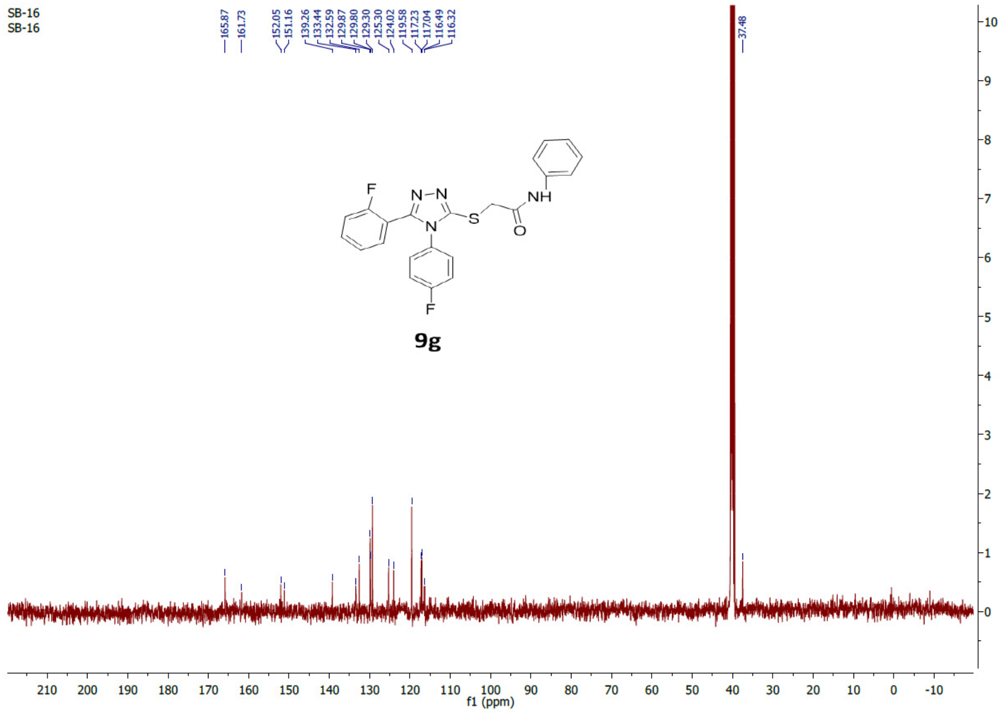

Figure S26, ES‡

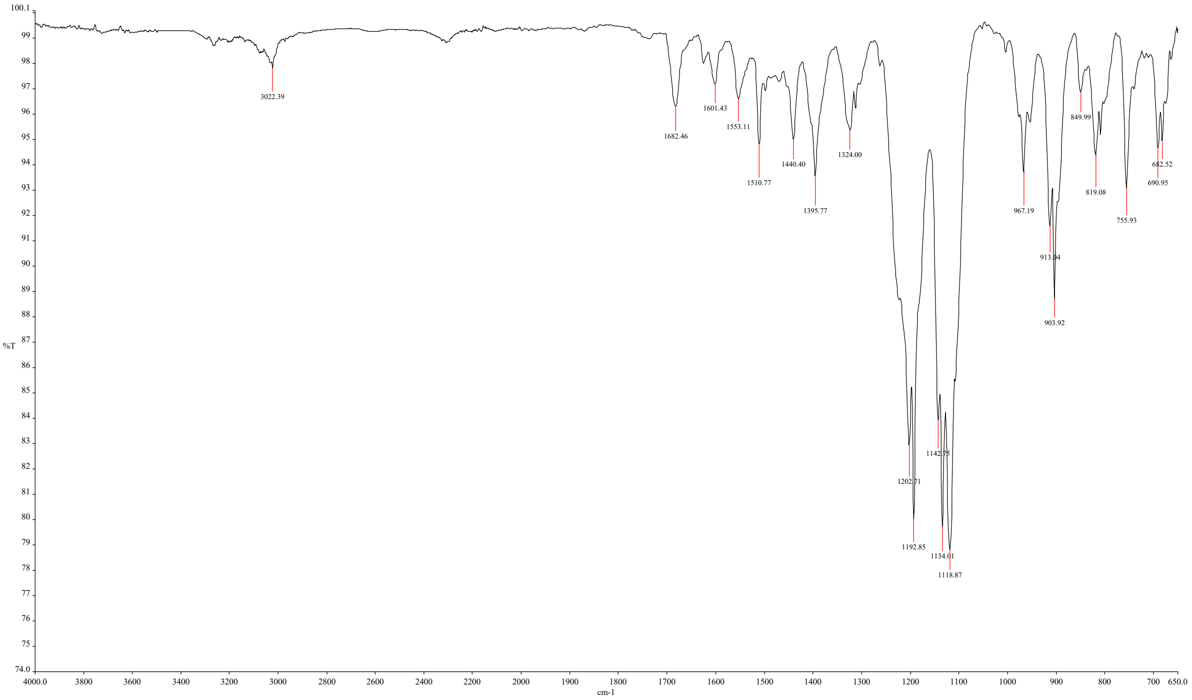

Figure S27, ES‡

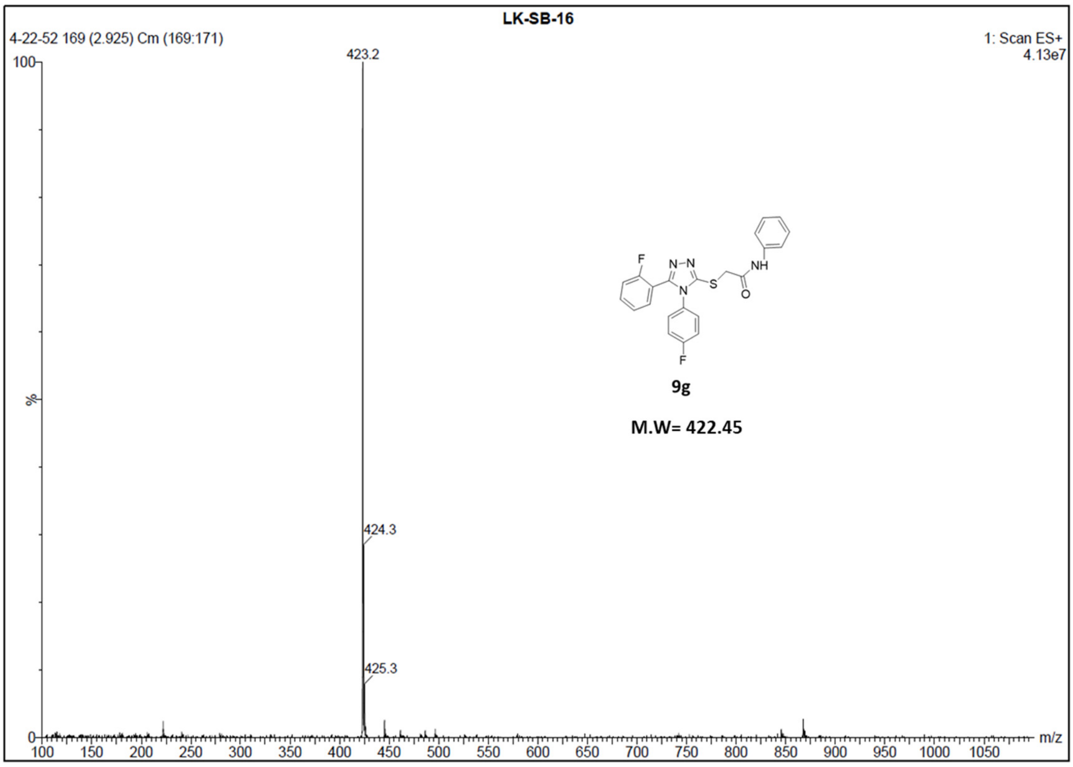

Figure S28, ES‡

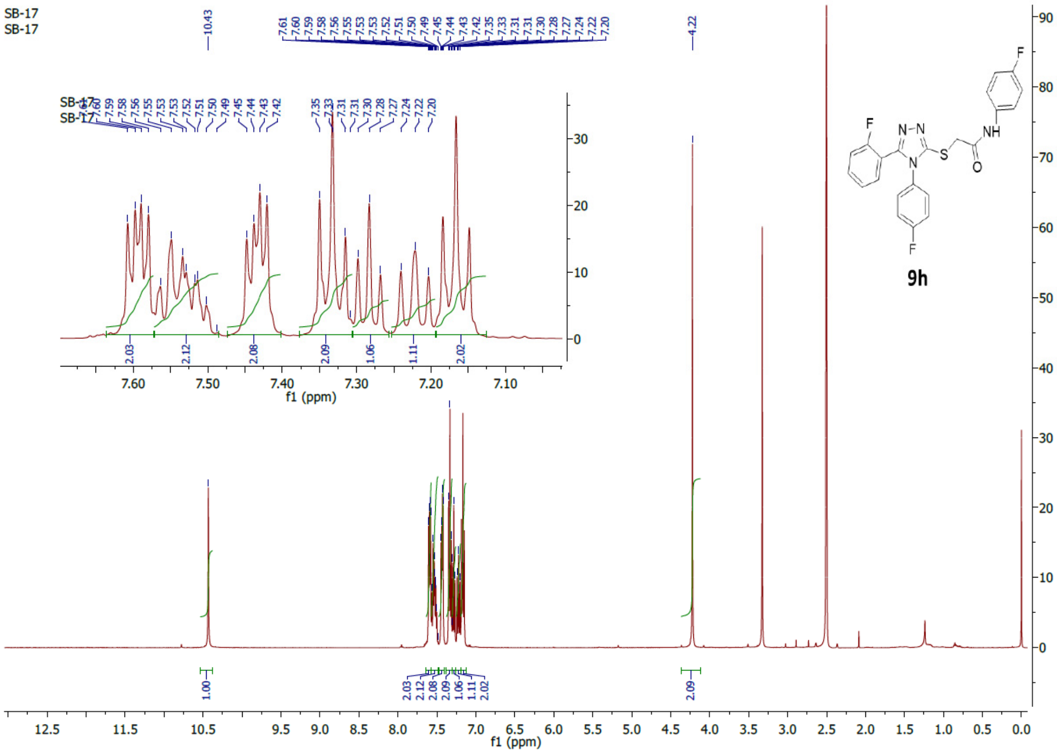

Figure S29, ES‡

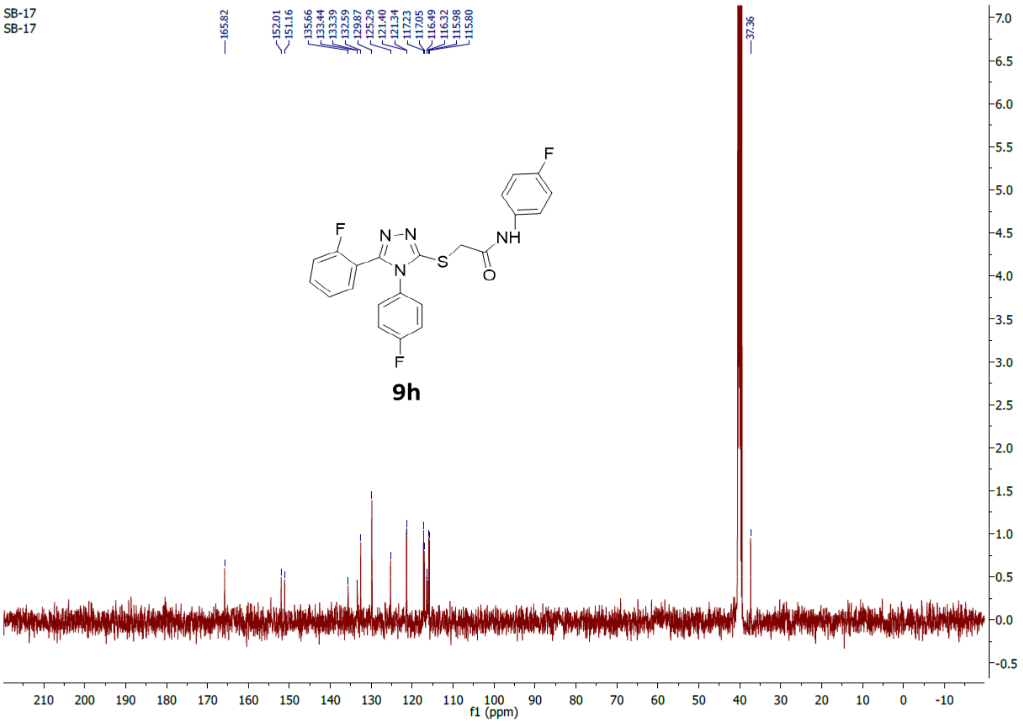

Figure S30, ES‡

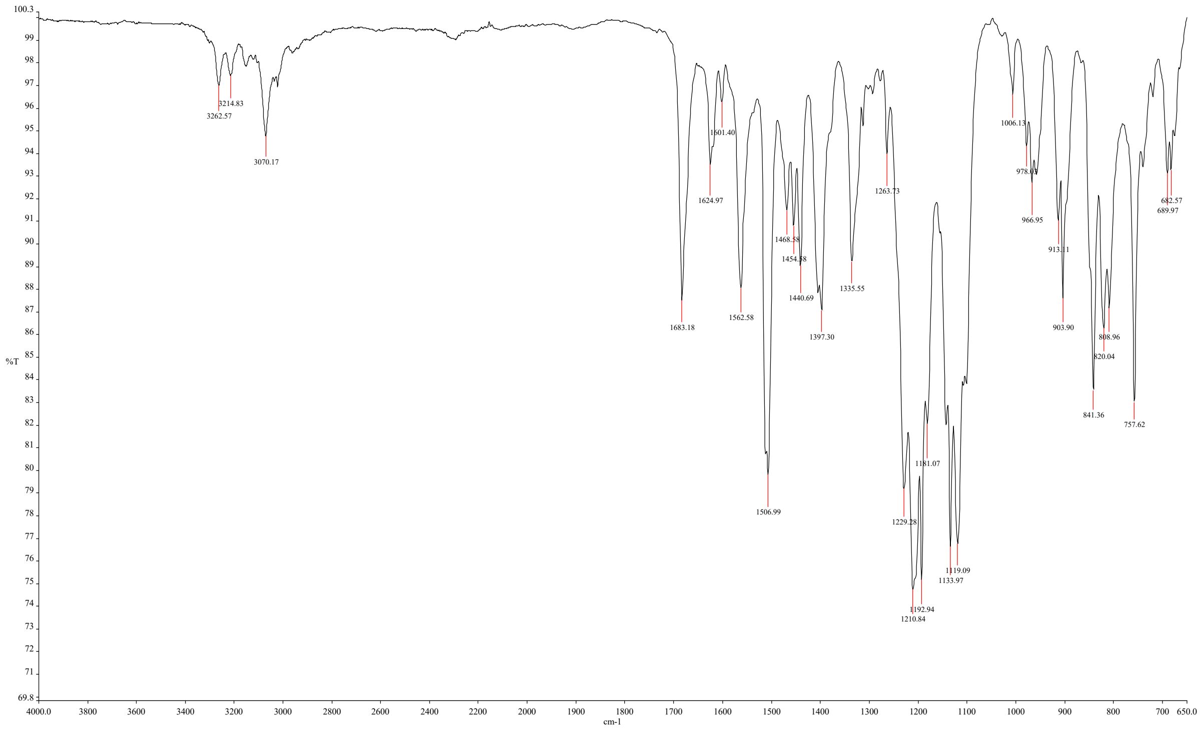

Figure S31, ES‡

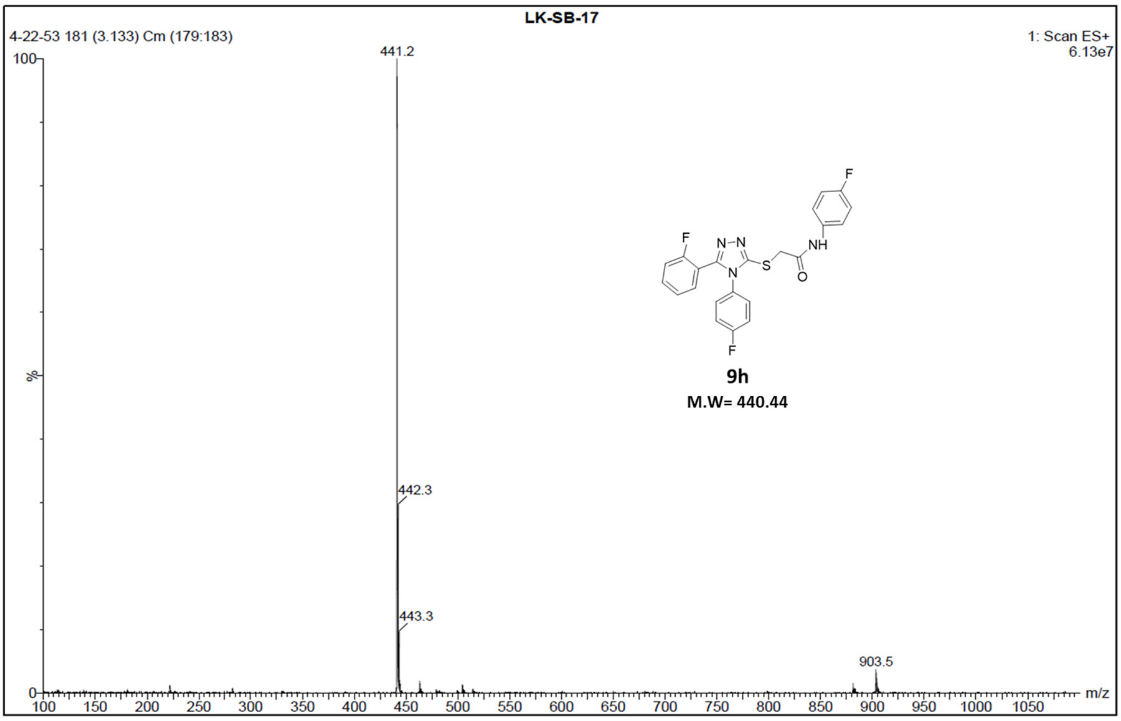

Figure S32, ES‡

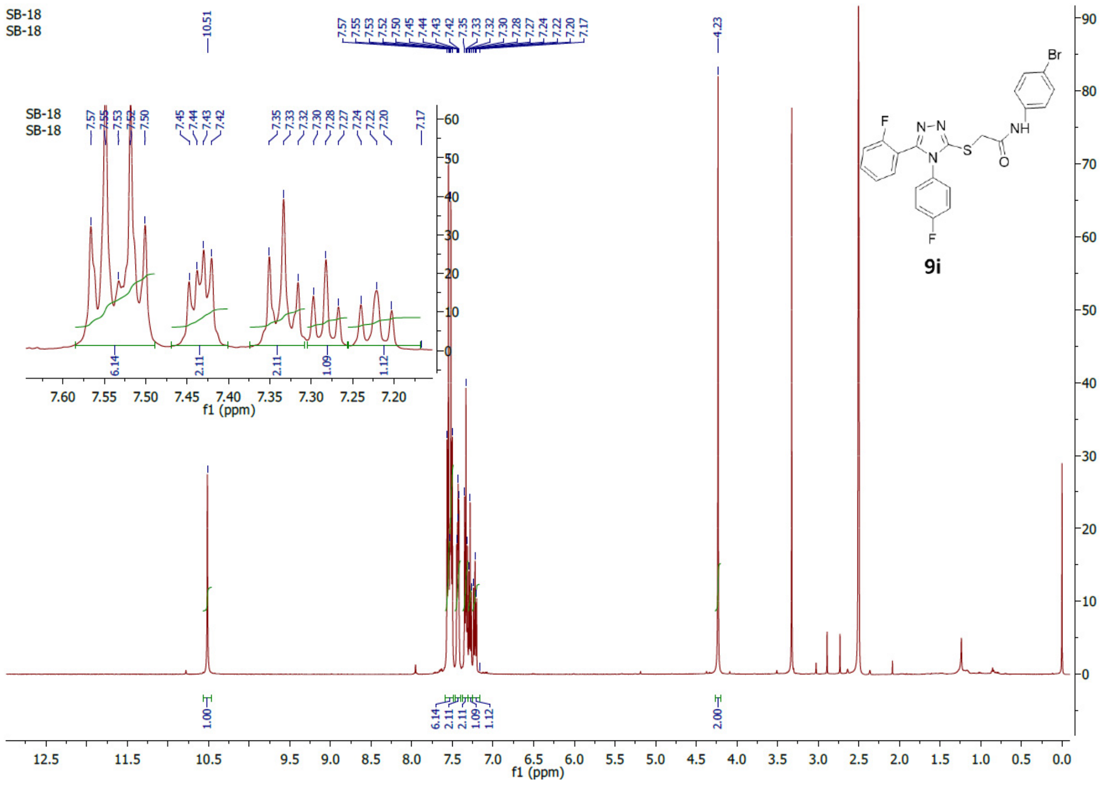

Figure S33, ES‡

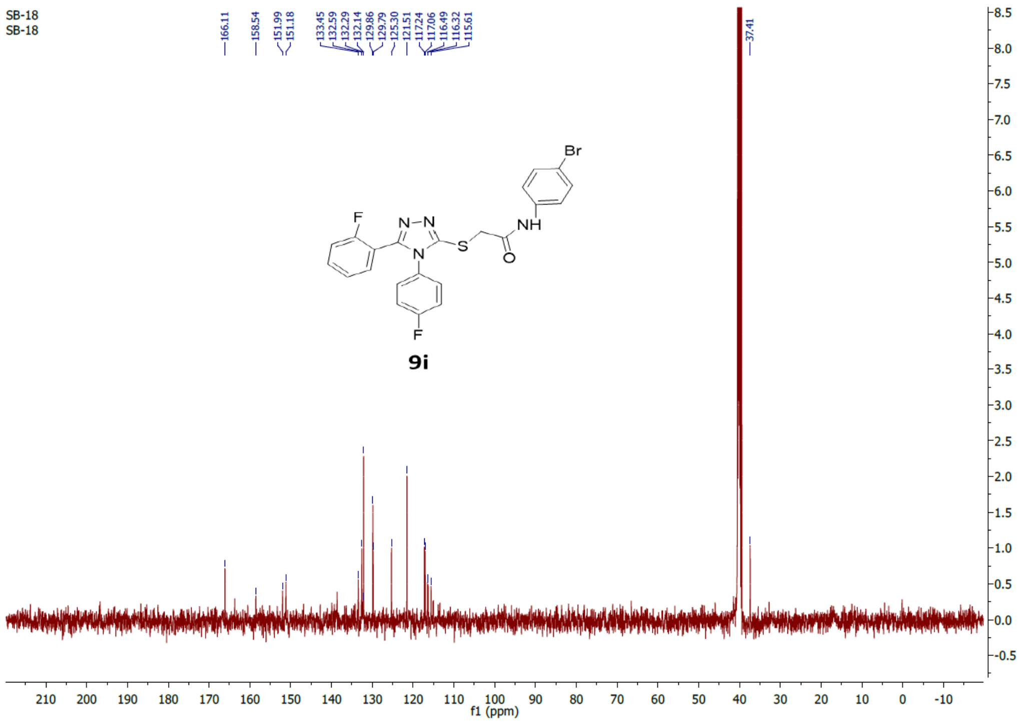

Figure S34, ES‡

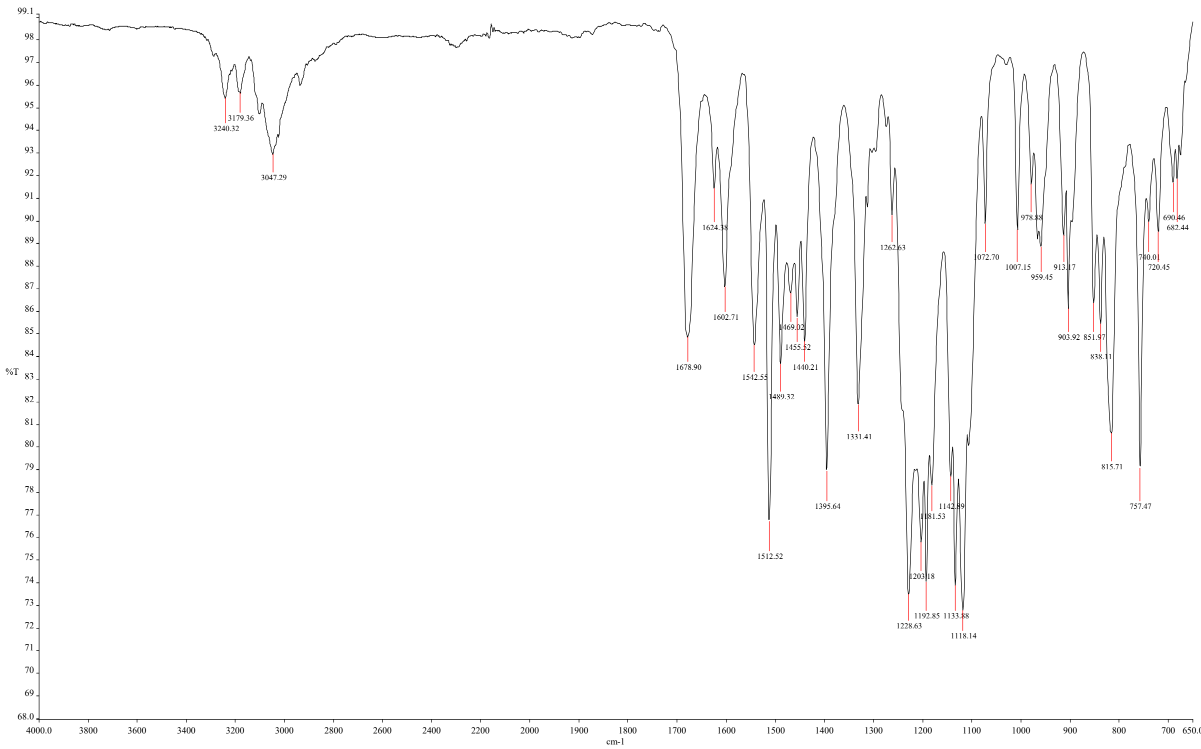

Figure S35, ES‡

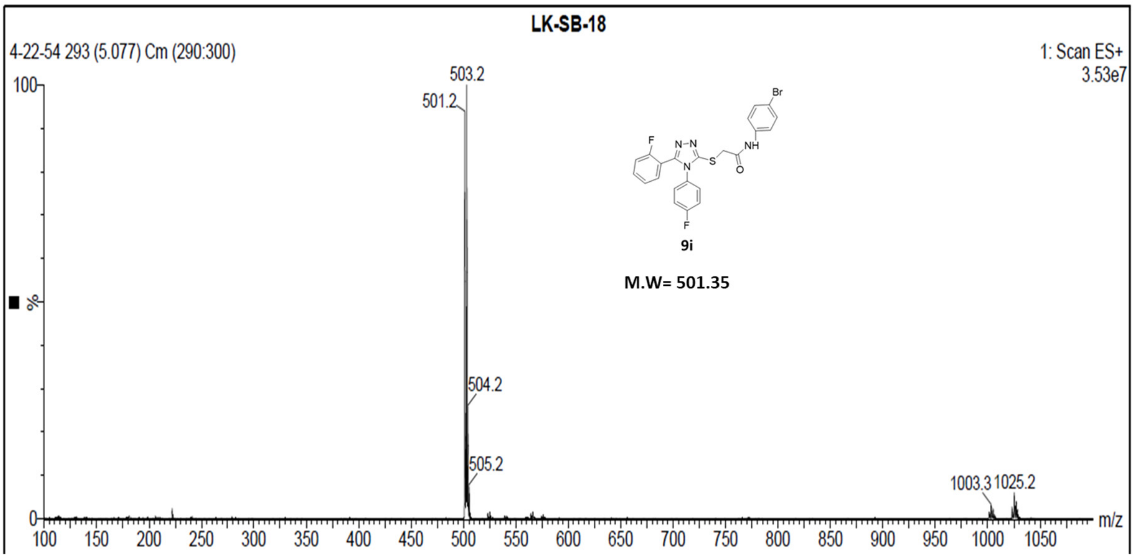

Figure S36, ES‡

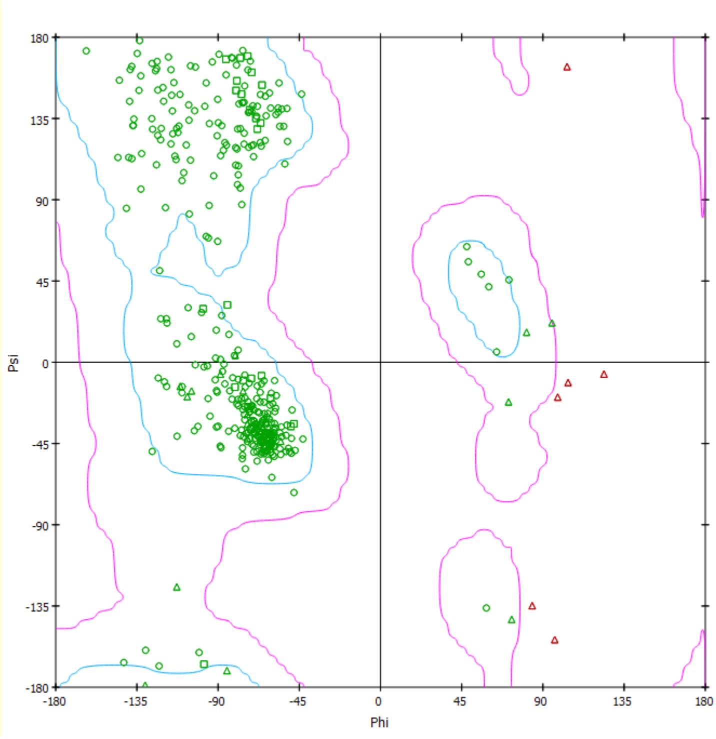

Figure S37, ES‡

9a

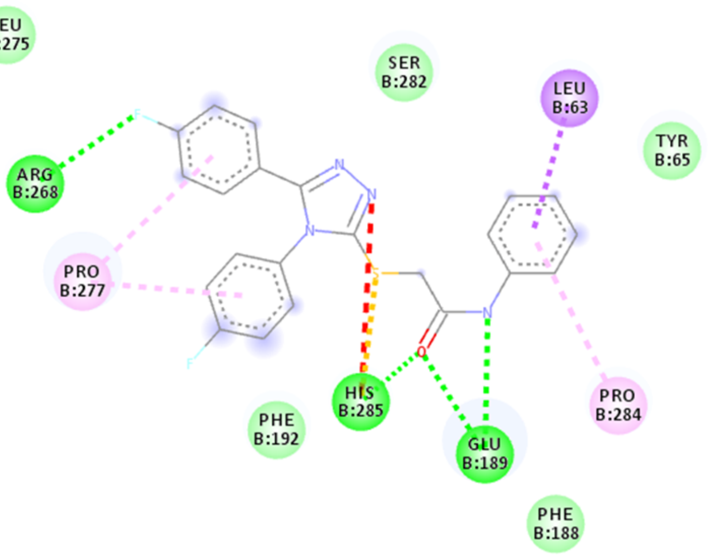

Figure S38, ES‡

9b

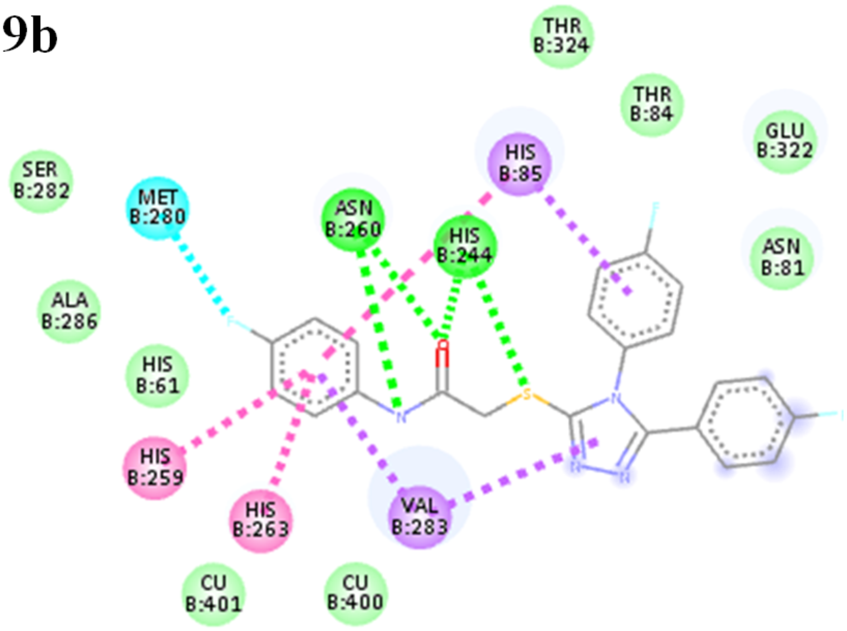

Figure S39, ES‡

9c

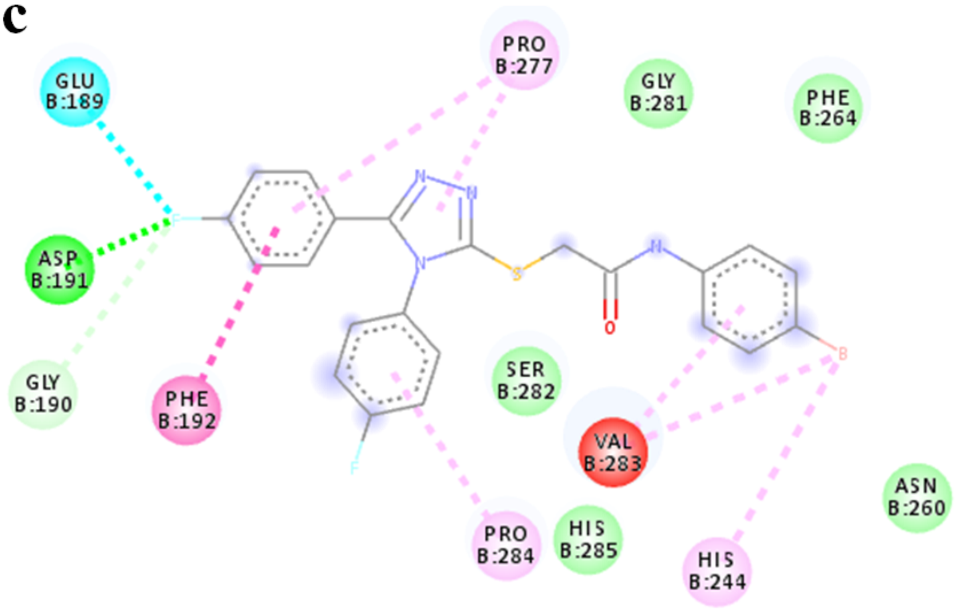

Figure S40, ES‡

9d

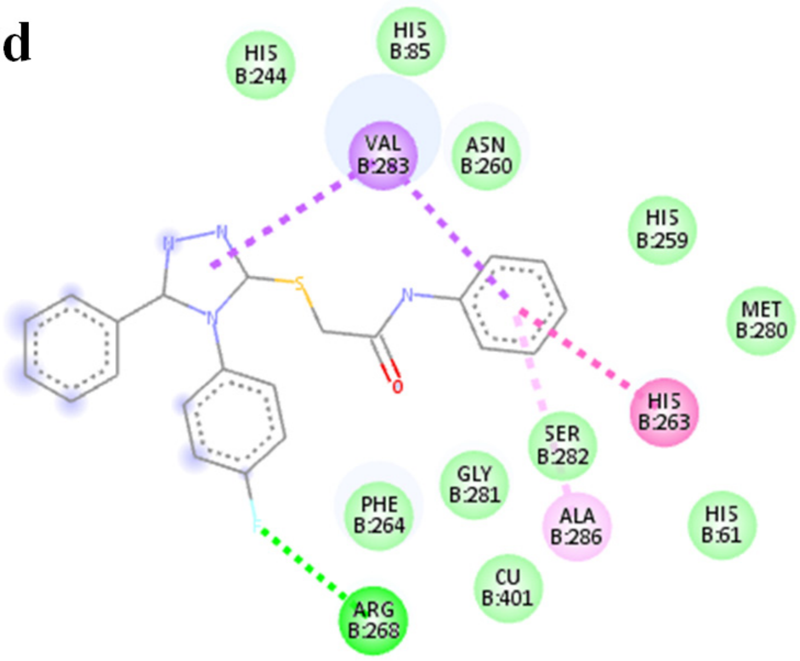

Figure S41, ES‡

9e

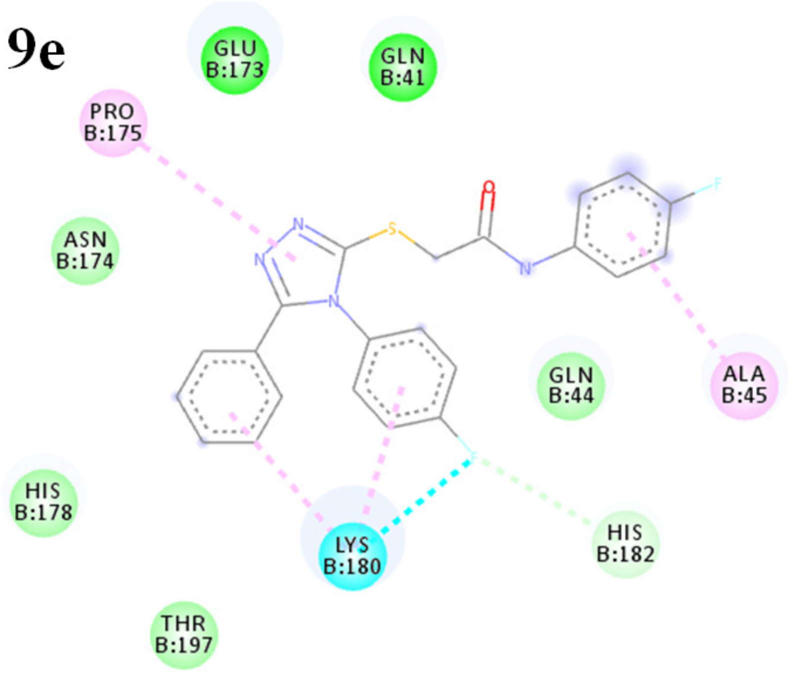

Figure S42, ES‡

9f

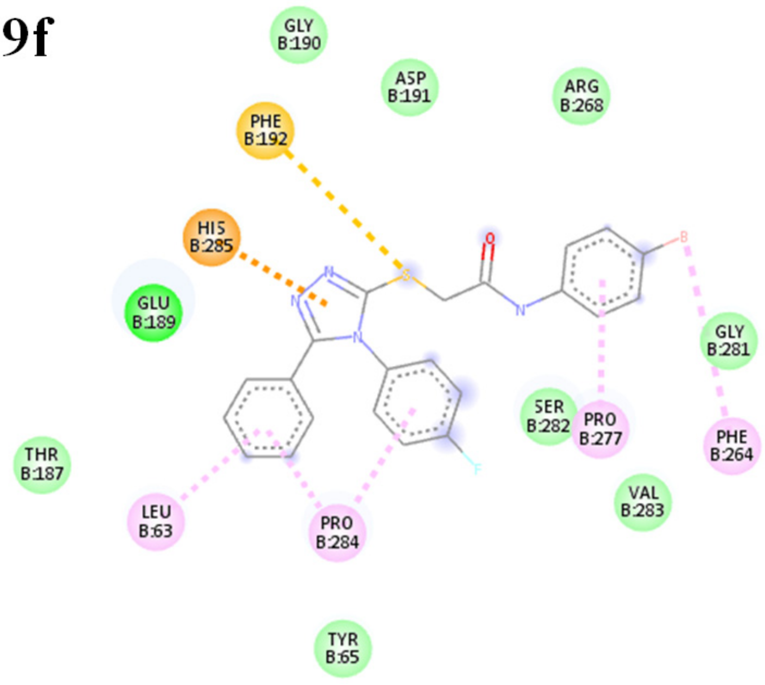

Figure S43, ES‡

9g

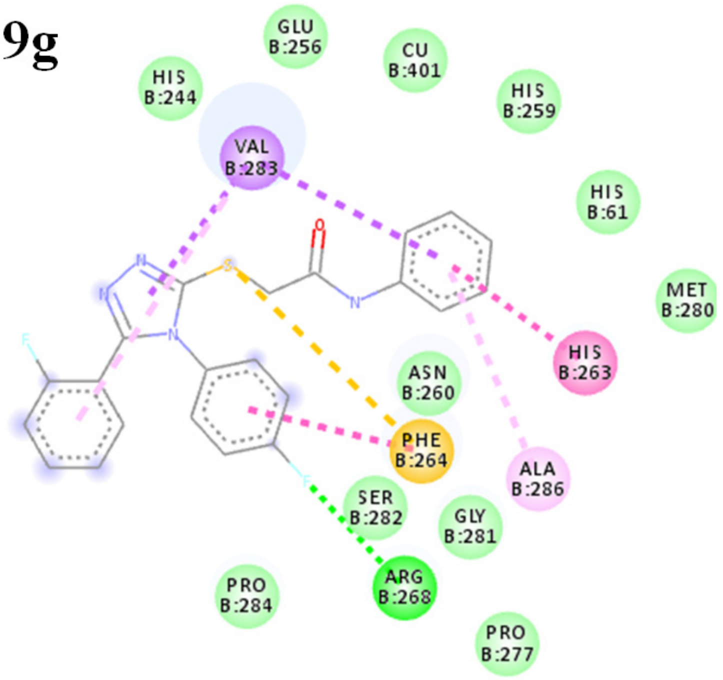

Figure S44, ES‡

9h

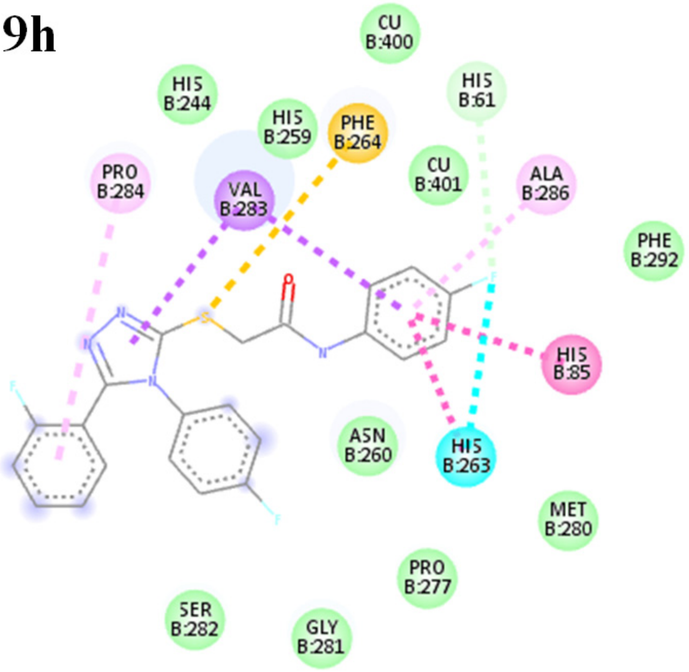

Figure S45, ES‡

9i

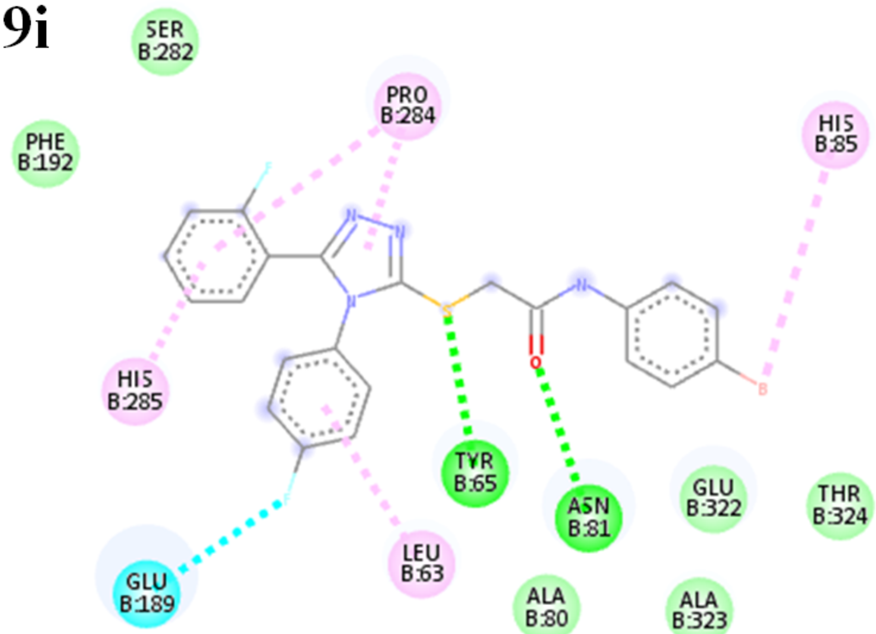

Figure S46, ES‡
